# Towards a physics-informed network paradigm with data generation and background noise removal for different distributed acoustic sensing applications

**DOI:** 10.1038/s41377-026-02295-5

**Published:** 2026-06-23

**Authors:** Yangyang Wan, Haotian Wang, Xuhui Yu, Jiageng Chen, Xinyu Fan, Zuyuan He

**Affiliations:** 1 State Key Laboratory of Photonics and Communications, Department of Electronic Engineering, Shanghai Jiao Tong University, Shanghai, 200240 China; 2 Ningbo AllianStream Photonics Technology Company, Ltd., Ningbo, China

**Keywords:** Optical sensors, Fibre optics and optical communications

## Abstract

Distributed acoustic sensing (DAS) has attracted considerable attention across various fields, and artificial intelligence (AI) technology plays a vital role in DAS applications for event recognition and denoising. Existing AI models require real-world data (RWD), whether labeled or not, for training, which is contradictory to the reality of limited available event data in practical scenarios. Here, a physics-informed DAS neural network paradigm is proposed, which eliminates the need for real-world event data during training. By physically modeling the target events along with real-world and DAS system constraints, physical functions are derived to train a generative network for the synthesis of DAS event data. A DAS noise-removal network is then trained using the generated data to effectively eliminate background noise in DAS measurements. The effectiveness of the proposed paradigm is demonstrated in two applications: event identification based on a public DAS spatiotemporal dataset, and belt conveyor fault monitoring based on DAS time-frequency data. In both cases, the paradigm achieves comparable or superior performance to data-driven networks trained with RWD. Owing to the incorporation of physical information and the ability to remove background noise, the proposed approach shows strong generalization capability across different sites within the same application. Notably, a fault diagnosis accuracy of 91.8% is achieved in a real belt conveyor field using networks transferred from a simulation test site, without using any fault event data from the target field during training. The proposed paradigm offers a potential solution to the critical challenges of limited data availability and intense noise in practical DAS applications.

## Introduction

Distributed acoustic sensing (DAS) offers long measurement range, high spatial resolution, high sensitivity and rapid measurements in real-time acoustic monitoring, leading to its extensive applications^1,2^. The DAS interrogator unit launches pulsed light into the optical fiber and receives the returning Rayleigh backscattering lightwave. By demodulating the phase changes of the Rayleigh backscattering lightwave, longitudinal strain caused by external perturbations at any position along the fiber can be acquired^3,4^. The high performance of DAS makes it a prospective solution for applications such as pipeline monitoring, geophysical exploration, structural health monitoring, seismic surveillance, and perimeter security^5–8^. In contrast to the excellent results achieved in laboratory simulation settings^9–11^, DAS may exhibit a high rate of false positives or false negatives, and may even fail to detect target event information in field applications. The common main challenges across various DAS applications lie in obtaining sufficient useful event data for signal analysis and extracting target event signals from severe environmental noise^6^. In recent years, artificial intelligence (AI) technology has developed rapidly and has been demonstrated to be applicable for DAS signal denoising, phase unwrapping, and event identification^12–16^. AI, with its powerful signal processing and analysis capabilities, offers promising solutions to the challenges encountered in practical DAS applications^17–20^.

The first critical challenge is the limited availability of event data^21^. Target events typically occur at only a few spatial locations and have relatively short durations. Consequently, the vast majority of the massive spatiotemporal data provided by DAS consists of background data, with only a limited amount of target event data. Furthermore, labeling target events in practice is not only time-consuming and labor-intensive, but also challenging in terms of ensuring label accuracy due to environmental noise and manual operation. Although many supervised neural networks have demonstrated excellent performance in DAS applications^22–25^, they typically require a large amount of high-quality labeled data for training, and the test data often shares the same experimental setup and environment as the training data. Semi-supervised learning and self-supervised learning neural networks have been explored to address the challenge of labeled data requirements^26,27^. Semi-supervised learning-based neural networks for DAS data processing have been employed to address the phase-picking task for earthquake monitoring^28^, conduct damage detection in pipes^29,30^, and perform track detection on high-speed railways^31^. A self-supervised learning fault classification algorithm with requirement of a small number of labeled samples is proposed for fault detection in belt conveyors^32^. By leveraging large amounts of unlabeled data, the classification network learns intrinsic features associated with faults. Unsupervised deep neural networks are utilized in geoscience to extract features from DAS spatiotemporal data for event category discrimination^33^. An adaptive decentralized artificial intelligence is proposed to improve the generalization performance of DAS algorithms by finetuning a pretrained model with unlabeled data from each site^34^. Although the above methods can effectively reduce or even eliminate the need for labeled data, they still require a sufficient amount of data that includes the target events for training. In applications such as structural health monitoring and seismic detection^35^, long-term data collection in real-world settings remains challenging due to the infrequent occurrence of target events. To avoid reliance on field data for training, a generative adversarial net (GAN) based DAS data generation network is proposed to provide simulated data to serve as a training dataset^36^. Real-world data (RWD) is still required to train the GAN, and a three-class classification network trained solely on simulated data achieves an accuracy of 64% when tested on experimental data. Using synthetic data as labeled data is another approach to network training. By employing finite difference modeling to simulate various microseismic events, simulated synthetic data acts as the training dataset for a neural network^37^. After training, an accuracy of 82% was achieved in binary classification on field data tests. It is worth noting that the network trained with seismic waveforms measured by traditional seismometers can be applied to DAS data for effective seismic wave detection^38^, indicating that both DAS and seismometer signals reflect similar physical processes.

The other key challenge is the environmental noise interference to the target signals in DAS applications^39^. Due to the high sensitivity characteristic of DAS, acoustic signals generated by non-target events in the actual environment are received, which constitute the environmental background noise with complex components and diverse attributes. Since conventional denoising algorithms struggle to address such complex noise, various machine learning- based DAS denoising algorithms have been explored^40,41^. The acquisition of training data, especially noise-free ground truth, for denoising networks is also a challenging or even impossible task. Benefiting from the high spatial density of DAS data, self-supervised learning methods can remove spatially incoherent noise with unknown characteristics in DAS data without the need for noise-free ground truth^42–44^. Furthermore, an improved blind-spot network has been proposed to remove noise exhibiting certain spatial correlations^45^. Although self-supervised methods reduce dependence on noise-free ground truth, noise typically needs to satisfy certain conditions, such as zero mean and a certain degree of independence. Moreover, self-supervised learning strategies may mistakenly identify background noise signals generated by environmental interference events as fault events in applications such as perimeter security and structural health monitoring. The key to effective background noise removal still lies in obtaining noise-free data of the target events. The utilization of synthetic data as noise-free data is an approach for the training of denoising neural networks. In traffic monitoring, an artificial algorithm is engineered to produce synthetic data of traffic trajectories, which are served as labels for the training of a denoising network^46^. In seismic wave monitoring applications, noise-free seismic data are generated by solving the elastic wave equation in many works, which are used as clean data for training denoising networks^47–49^. It is worth noting that the distinctive features of differential phase signals from DAS outputs are not considered in current synthetic data schemes for seismic monitoring. Overall, most existing research relies on real-world data, with insufficient consideration given to the physical characteristics of DAS data across various applications. Although synthetic data schemes have been demonstrated for noise reduction and event identification in seismic wave monitoring due to mature theoretical models, most DAS applications in complex scenarios lack methods for establishing physical models of DAS data. This limitation hinders the broader adoption and application of DAS across diverse fields.

In this work, we present a physics-informed neural network paradigm capable of generating DAS data and removing background noise for deployment in different DAS applications without needing real-world event data. The proposed paradigm firstly utilizes physical functions to train the physics-informed generative network (PIGN) to generate a large amount of DAS event data, which solves the challenge of obtaining DAS event data. The physical functions consist of the physical model and expert experience corresponding to the event, as well as the constraint functions of the real world and DAS system. By combining the generated event data with easily obtainable real-world normal background data, a DAS noise-removal net for the elimination of intense background noise is trained. After training the classification network with denoised normal data and generated event data, it can be applied in the field for event recognition. The proposed paradigm is validated on different DAS applications. In an event recognition task, DAS data of shake and walk events are generated by PIGN and compared with RWD from the public dataset. The classification performance of networks trained with generated data is comparable to that of networks trained with RWD. Since the public dataset exhibits low environmental noise, the background noise-removal network was not employed. The belt conveyor fault monitoring application is selected due to the presence of significant background noise. At the simulation test site, the denoised data from the noise-removal net show better classification performance than the initial data across various classification networks, due to the effective removal of intense background noise similar to event signals. Attributed to the introduction of physical knowledge, the network paradigm shows generalization capability and can be quickly deployed at different sites for similar DAS applications. After transferring the network paradigm from the simulation test site to the field, a belt conveyor fault diagnosis accuracy of 91.8% on field data without any real-world event data for training is achieved, which is higher than the results of 82.2 and 86.6% for the conventional data-driven neural network and artificially designed algorithm based on real-world fault signals.

## Results

### Physics-informed DAS neural network paradigm

**Fig. 1:**
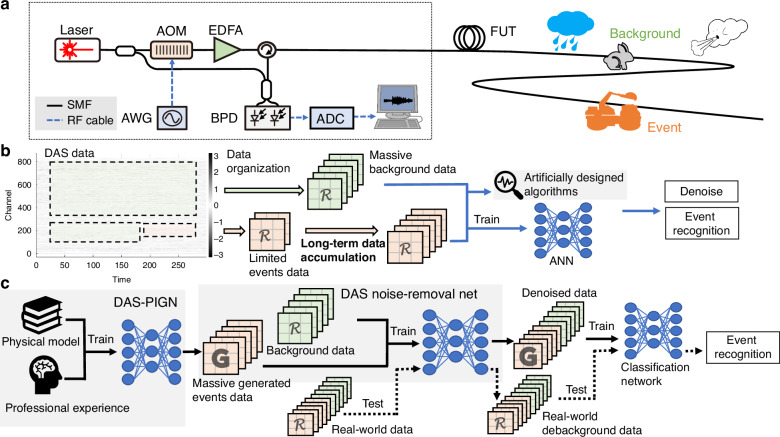
Schematic of the proposed physics-informed DAS neural network paradigm. **a** Schematic diagram of the DAS system and its application. SMF single-mode fiber, RF radio frequency, AWG arbitrary waveform generator, AOM acousto-optic modulator, EDFA erbium-doped fiber amplifier, BPD balanced photodetector, ADC analog-to-digital converter, FUT fiber under test. The DAS system simultaneously detects both background and event signals in practical applications. **b** Traditional design process of DAS signal processing algorithms. ANN artificial neural network. A long-term data accumulation process is required to obtain a sufficient amount of event data, whether labeled or not. **c** Physics-informed DAS neural network paradigm. PIGN physics-informed generative network. The training of PIGN, noise-removal net, and classification network in the network paradigm does not require any real-world event data. PIGN is used to generate data, the noise-removal net is used to remove background noise, and the classification network is used for event recognition

The experimental setup and application deployment of the DAS system are shown in Fig. 1a. In DAS, the laser is modulated into pulsed light and injected into the fiber under test (FUT). The location of any point along the FUT can be determined according to the round-trip time of pulsed light in the FUT. The Rayleigh backscattering light field generated on FUT interferes with the local light field to generate an interference signal. Since the phase change of the backscattering light field is linear with the strain on the optical fiber, the quantitative measurement of an acoustic wave can be achieved by acquiring phase information from the interference signal. When DAS is applied in field applications, the acoustic waves of target events and background interference events are received simultaneously, which makes it difficult to extract and analyze the target signals. For most DAS applications, the occurrence of target events is rare, such as seismic wave detection, structural health monitoring and pipeline monitoring. Therefore, the vast majority of DAS data consists of background data containing various environmental noises due to the high spatiotemporal density characteristic of DAS data. This leads to the requirement of a long-term data accumulation process to obtain a large number of target event data for the design of artificially designed algorithms or the training of conventional data-driven neural networks for DAS data processing, as shown in Fig. 1b. The data accumulation process may encounter difficulties when the application environment is complex and the background noise is intense.

In order to overcome the challenges of data accumulation process and significant background noise in DAS applications, a physics-informed neural network paradigm, including DAS data generation and denoising is proposed as shown in Fig. 1c. DAS physics-informed generative network (PIGN) is first proposed to produce massive generated event data. PIGN only needs to be constrained by the physical model of the target event in the training process, without the requirement of any RWD. For events that are difficult to be physically modeled, professional experience can be converted into empirical functions as training constraints for PIGN. The generated data can be regarded as noise-free target event signals. Using these generated noise-free ground truths, a DAS noise-removal net is proposed to eliminate complex environmental noise. The training dataset of the noise-removal net is composed of generated event data and easily obtainable real-world background data. Since the background environment is unique in different DAS application scenarios, it is difficult and unnecessary to physically model the background data. In practical DAS applications, DAS signals at most spatial locations belong to the background data, which is easy to obtain. Given the characteristics of DAS background data, the real-world background data should be directly collected for data analysis. The core purpose in most DAS applications is event recognition. Hence, a neural network for event classification is trained using generated event data and real-world background data. The proposed DAS data processing network paradigm does not require real-world event data during the entire training process. Once the noise-removal net and classification network have been trained, they can be directly applied to RWD for environmental noise removal and event recognition in the field.

### Physics-informed generative network

**Fig. 2:**
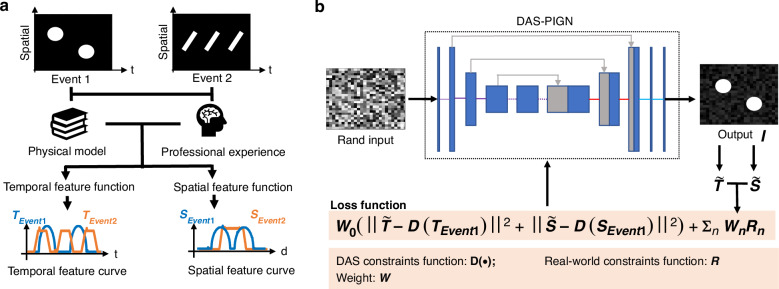
Principle of PIGN. **a** Schematic of feature functions acquisition for DAS event data. After projecting DAS event data onto different dimensions, and combining physical models with professional experience, the corresponding feature functions of the events in the respective dimensions can be obtained. Feature curves of an individual event can then be derived from these feature functions. **b** Network structure and training process of PIGN. Network is based on the U-Net architecture. During training, the loss function is defined by assessing the gap between the feature curves of the output image and the target feature curves, while also incorporating constraints from the DAS system and real-world conditions

The proposed framework does not require a fully precise physical model to operate effectively. However, its performance improves as the discrepancy between PIGN-generated data and RWD becomes smaller. The working principle of PIGN is shown in Fig. 2. Signals generated by different types of events often possess distinct spatiotemporal features. Since DAS data is two-dimensional, comprising temporal and spatial dimensions, it is capable of recording features of various events. The spatial and temporal features of an event can be delineated by viewing DAS data as an image and projecting curves along the respective axes, as shown in Fig. 2a. Typically, the temporal and spatial feature functions of a class of events can be derived from theoretical physical models based on research in related fields. For applications where the theory is underdeveloped or difficult to model due to the complex circumstances, the temporal and spatial feature functions can be derived from experiential knowledge of the expert. Using these feature functions, a large number of feature curves can be obtained to depict multiple cases of the event. PIGN is based on a U-Net structure, taking random data as input and generating event data as output. In PIGN, the temporal and spatial feature curves of the event are used as target curves for network training, as shown in Fig. 2b. The output data from the network are processed to obtain the predicted feature curves, which are then compared with the target curves using mean squared error as part of the loss function. Although high-quality image reconstruction often requires curve projections from multiple directions^50^, projection curves along the time and space dimensions have provided sufficient information for PIGN to learn the spatiotemporal features of the event.

To narrow the gap between generated data and RWD, constraint functions based on real-world characteristics and the DAS system have been incorporated into the loss function. The design of the real-world constraint function must be tailored to the specifics of the target application. Since real-world signals are predominantly continuous in the time domain, the average of the squared temporal differences in the generated DAS signal is calculated and incorporated as a type of real-world physical constraint function *R* into the loss function, which can be expressed as:

1 $$\frac{1}{n-1}\mathop{\sum }\limits_{i=1}^{n-1}{(\Delta {I}_{i})}^{2}$$

where, *n* is the total number of samples in the time dimension. Note that *I* still denote the output image. With this continuous-signal constraint function, unreal spurious spikes in the generated data can be eliminated. In many scenarios, correlations in DAS data can be observed across multiple channels within dimensions such as space, time, and frequency. Taking the spatial dimension as an example, the spatial-correlation constraint function added to the loss function as another type of real-world constraints function can be expressed as:

2 $${(\mathop{\sum }\limits_{i}\mathop{\sum }\limits_{j}{C}_{{s}_{i}{s}_{j}}-{C}_{T})}^{2}$$

where ${C}_{{s}_{i}{s}_{j}}$ is the correlation coefficient between channel *i* and *j*, *C*_*T*_ is the target value of the total correlation coefficient. Continuous signals and correlation constraint functions in other dimensions can be similarly constructed based on the characteristics of the event. In the applications presented below, the continuous-signal constraint and the spatial-correlation constraint serve as the real-world constraint functions. In addition to these constraints, the DAS instrumentation itself also introduces characteristic influences on the measured signals. Since phase wrapping is a common phenomenon and an important characteristic in DAS data, a phase wrapping algorithm should be applied to the objective feature functions as a type of DAS constraint function. Taking the temporal feature function *T* as an example, the phase wrapping constraint can be expressed as:

3 $$D(T(t))=T(t)-2\pi \left[\frac{T(t)+\pi }{2\pi }\right]$$

where $[.]$ is the floor function. Details about the real-world and DAS system constraint functions are provided in the supplementary material. By using the objective feature functions as learning targets and incorporating constraints from both the real world and the DAS system, PIGN can synthesize large amounts of DAS data for target events. Therefore, the full loss function of the PIGN network is expressed as:

4 $${W}_{0}({\Vert \tilde{T}-D({T}_{Event})\Vert }^{2}+{\Vert \tilde{S}-D({S}_{Event})\Vert }^{2})+\mathop{\sum }\limits_{n}{W}_{n}{R}_{n}$$

where *D*() is the DAS constraints function and *R* is the Real-world constraints function. *W* represents the weight. The specific functional forms of *D*() and *R*, as well as the number of *R* terms, may vary depending on the application scenario. We adopted an untrained mode for training, and details are in the supplementary materials.

### Event recognition with PIGN

The effectiveness of PIGN is validated on a public DAS dataset through the event recognition task^24^. The public DAS dataset includes training and testing datasets for six distinct event classes, with each class having roughly 2000 training and 500 testing samples. The background, shake, and walk data are selected to validate the PIGN. Although the public dataset contains six event types, our expertise does not allow us to develop physical models for the remaining three. This underscores the importance of collaborating with domain experts who are capable of establishing the corresponding physical models.

**Fig. 3:**
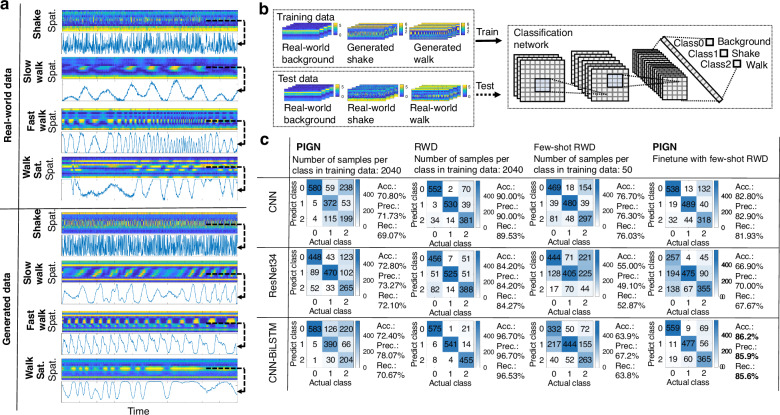
Event recognition performance on a public dataset. **a** Examples of shake and walk DAS data from the public dataset and PIGN-generated dataset. Spat. spatial, Sat. saturation. PIGN can generate data that possesses the characteristics of signal saturation and phase wrapping observed in practical applications. **b** Flowchart of the training and testing process for the classification network. The training dataset comprises only easily accessible real-world background data and event data generated by PIGN, with no real-world event data included. **c** Event recognition results in different networks with the same real-world test data from a public dataset. RWD real-world data; Acc. accuracy, Prec, precision, Rec. recall. class 0: background; class 1: shake; class 2: walk. PIGN means training with generated event data, whereas RWD means training with real-world event data

Shaking can be described by a forced vibration model, while walking conforms to a pedestrian motion model (see Methods). Based on these physical models, combined with the model of DAS signal generation mechanisms, both temporal and spatial feature functions are derived for these two classes of events. Using these objective feature functions, PIGN can generate corresponding DAS event data, which is compared with RWD as shown in Fig. 3a. The generated data and RWD present similar structural patterns. For the public dataset used here, it is not specified whether the measurements correspond to differential phase or absolute phase. In our applications, however, the temporal feature models are typically constructed from sinusoidal functions, whose general form is preserved under integration or differentiation: the characteristic frequency of an event remains unchanged, and only the associated coefficients vary. Consequently, even without knowing whether the dataset reflects differential or absolute phase, the physical model can still capture the essential event characteristics. This indicates that the proposed PIGN approach does not require an exact correspondence between the physical model and the measured DAS signals; it suffices that the model represents the fundamental features of the underlying events.

Owing to the integration of the DAS system and real-world constraint functions, the generated data is capable of simulating signal saturation and phase wrapping. As shown in Fig. 3b, the generated event data and real-world background data are used as training data for training the classification network. The reason for using real-world background data is that it is easily accessible in practical DAS applications. After training, RWD is used for testing the model. Multiple classification networks were trained with varying amounts of RWD under the same network structure and parameters to compare with classification networks trained on generated data, as shown in Fig. 3c (statistical evaluation in Supplementary Materials). Three representative basic classification networks, convolutional neural network (CNN)^51^, residual network (ResNet)^52^, and convolutional neural network bidirectional long short-term memory (CNN-BiLSTM)^53^, were trained to demonstrate that the validity of the generated data is independent of the network architecture. The classification accuracies of the three types of networks trained with PIGN output data are above 70%, surpassing the 55% and 63.9% accuracies of ResNet and CNN-BiLSTM networks trained with 50 samples of RWD per class. To ensure reliability, the experiment of randomly selecting a small amount of RWD from the public dataset for network training was repeated 100 times. Figure 3c shows the result of one trial with relatively high accuracy (see Supplementary Materials for details). Networks initially trained with PIGN data were further finetuned using a small RWD dataset with 50 samples per class. This process led to accuracy improvements of 6.1, 11.9, and 22.3% for the CNN, ResNet, and CNN-BiLSTM networks, respectively, compared to networks trained on the same small RWD dataset. The finetuned CNN-BiLSTM reached an accuracy of 86.2%, exceeding the performance of ResNet when trained on a large dataset of RWD. All results in Fig. 3c were subjected to statistical significance testing, which shows that PIGN-Finetune and few-shot RWD exhibit a statistically significant difference. PIGN-Finetune consistently achieves superior performance, highlighting the effectiveness of the PIGN-generated data. The results demonstrate that the data generated by PIGN can effectively simulate RWD, and the classification network trained with generated data has comparable or superior performance to the networks trained with a limited amount of RWD. Compared with results trained with large amounts of RWD, the lower accuracy with PIGN-generated data is reasonable because the simplified physical models and broadly chosen parameters cannot fully reproduce the complexity of specific real-world scenarios. Moreover, the public dataset contains only subtle differences between its training and testing conditions, which inherently benefits models trained on real-world data.

### Noise-removal net

Generated data from PIGN can also be utilized to train a DAS signal denoising network, addressing the challenge of severe environmental background noise interference in DAS applications.

**Fig. 4:**
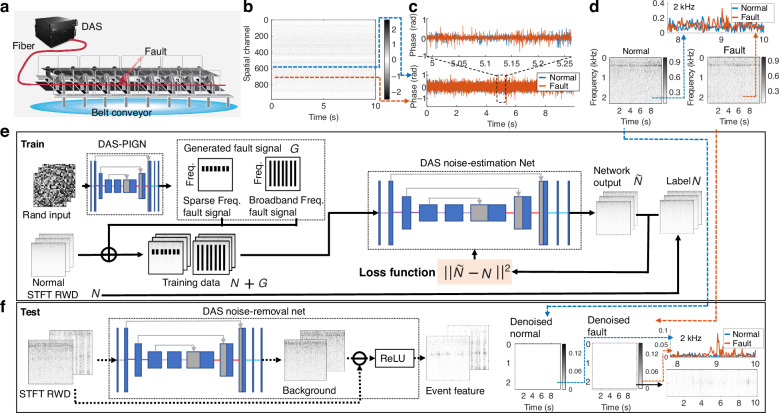
Schematic of the DAS noise-removal net. **a** Schematic illustration of belt conveyor fault monitoring. **b** Example DAS data in belt conveyor monitoring. **c** Temporal DAS signals for normal and fault states in the belt conveyor. **d** DAS time-frequency diagrams for fault and normal state. The zoomed-in figure depicts the temporal variation of signals at 2 kHz for both fault and normal states. Due to the presence of intense background noise signals, it is difficult to distinguish normal and fault signals in both time and time-frequency domains. **e** The training process of DAS noise-removal net. STFT short-time Fourier transform. The DAS noise-removal net is based on the U-Net structure. **f** Testing process of the DAS noise-removal net. ReLU, rectified linear unit. After denoising, the fault signal can be distinctly observed at 2 kHz

Although end-to-end learning methods do not require a separate denoising network, some studies have shown that a specially trained denoising network can effectively improve the performance of various downstream tasks^54,55^. More importantly, the PIGN network cannot reproduce complex background noise and can only generate relatively idealized event signals. Consequently, the classifier trained on PIGN-generated data requires input with minimal noise. Therefore, in our proposed paradigm, denoising is necessary before the final downstream task. Since the public dataset was collected in a laboratory environment where background noise is not strong enough, we chose the application of belt conveyor fault monitoring, as shown in Fig. 4a, which involves significant environmental noise. Figure 4b shows typical DAS data in the temporal and spatial dimensions for belt conveyor monitoring (an enlarged version is provided in the Supplementary Material). Mechanical vibrations accompany the normal operation of the belt conveyor, causing significant background noise in DAS data. Due to the intense background noise and intermittent fault signals characteristic of belt conveyor monitoring applications, time-frequency DAS data were obtained by applying the short-time Fourier transform (STFT) to the time-domain DAS data at each spatial channel, enabling analysis of the signal’s time-varying frequency information. For less severe faults, the relatively weak fault signals are often overwhelmed by the background noise, making it difficult to distinguish fault signals from normal signals in both the time and time-frequency domains, as shown in Fig. 4c, d. Moreover, the vibrations from normal operation share similar mechanisms with the vibration induced by some types of faults, resulting in background noise in the normal signal has similar features with the fault data. Therefore, it is necessary to perform denoising to extract the target event signal.

**Fig. 5:**
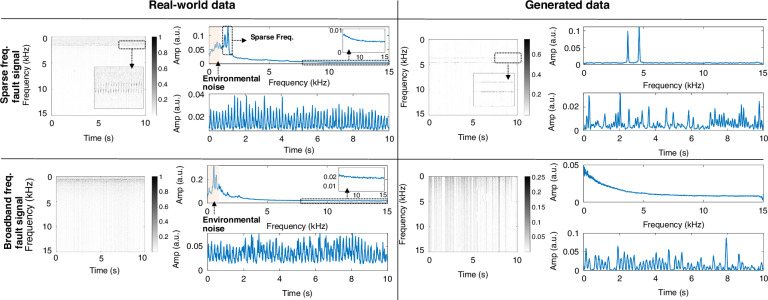
Examples of fault signals in belt conveyor from real-world data and PIGN-generated data. Real-world data with intense fault signals are presented for clear demonstration, and show periodic impulsive features with varying intensities in the time domain. Due to strong low-frequency environmental noise during belt conveyor operation, both sparse frequency and broadband frequency faults in the real-world data have strong energy below about 1 kHz. The main difference is that sparse frequency faults show clear, strong frequency components above 1 kHz but low intensity in other bands, while broadband frequency faults have no obvious strong components beyond the low-frequency noise and maintain a certain intensity across the entire frequency range. PIGN-generated data possess characteristics similar to real-world data

The training and testing processes of the proposed DAS noise-removal net are shown in Fig. 4e, f. The first step is to obtain data generated by PIGN of fault events for training. In the belt conveyor fault monitoring application, building a theoretical physical model is very challenging due to the complex environment. Therefore, in this case, the objective feature functions used to train PIGN are established based on experiential knowledge (see Methods). Considering that each roller in the belt conveyor corresponds to a specific spatial position, and the signals from different rollers are nearly independent, the objective feature functions describe the time-frequency domain of the data at a single position. According to expert experience and related literature^56^, roller faults can be roughly divided into two types: sparse frequency and broadband frequency fault signals. The corresponding fault data generated by the trained PIGN are shown in Fig. 5 (more results in the Supplementary document). These generated fault data were then combined with real-world normal data to train the noise-removal network. After training, the noise-removal network can be used to extract background noise from the RWD. Subtracting the network output linearly from the input RWD gives the background-removed event feature signal, as shown in Fig. 4f. The ReLU operation is applied to ensure that the resulting event features do not contain meaningless negative values (see Supplementary Materials for details). The noise-removal net adopts a residual learning structure, focusing on learning the background data rather than directly extracting event features (see Methods). The main reasons are the accessibility of a substantial amount of real-world background signals, and the fact that low-frequency components within the background noise are more easily captured by the network, following the frequency principle in neural network training^57^. As shown in Fig. 4d, f (enlarged version in Supplementary Materials), after the background noise is removed by the noise-removal net, the subtle fault signal at a frequency of 2 kHz can be distinctly observed. Before background noise removal, the fault signal at 9 s is difficult to distinguish due to its comparable amplitude to background noise at other time points. After background noise removal, the fault signal at 9 s becomes clearly identifiable, with an amplitude at least twice as large as that at other time points.

**Fig. 6:**
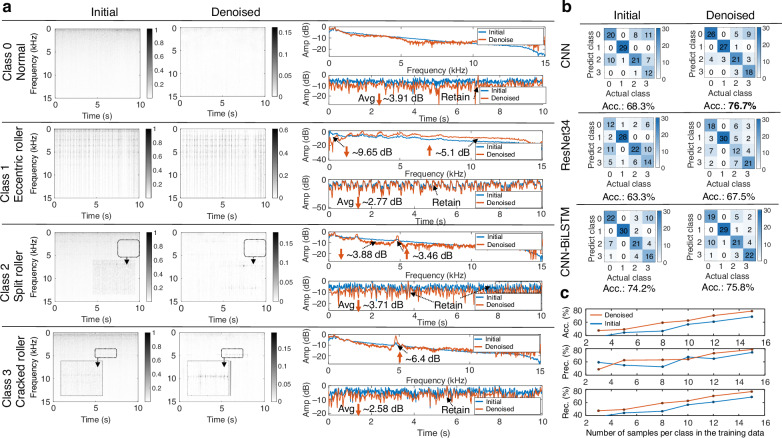
Denoised results from the noise-removal net and fault diagnosis results in the belt conveyor simulation test site. **a** Comparison of initial and denoised data for different classes. The noise-removal net can eliminate background noise while retaining fault signals with sparse or broadband frequency features. **b** Fault recognition results using initial and denoised data. Denoised data can achieve higher classification accuracy in various networks. **c** The classification performance of initial and denoised data in CNN under different training datasets with varying sample sizes. Compared to the initial data, the denoised data facilitates higher classifier accuracy

The experiment was conducted at the belt conveyor simulation test site provided by Ningbo AllianStream Photonics Technology Co., Ltd, to acquire DAS data of normal rollers, eccentric rollers, split rollers, and cracked rollers (see Methods). The initial time-frequency data and the background removal results by the noise-removal net for the four classes are shown in Fig. 6a. The temporal and frequency feature curves are obtained by calculating the average energy in their respective domains. For the convenience of comparison, both the initial and denoised feature curves have been normalized individually. After denoising, the maximum amplitude of the normal data decreased from 1 to 0.15, primarily retaining an abnormal feature at 7 s. The frequency feature curve of the normal data after denoised changed little, and the temporal feature curve showed an approximately average decrease of 3.91 dB. Fault data from eccentric rollers, which exhibit broadband frequency features, as well as data from split and cracked rollers with sparse frequency features, were effectively denoised by the noise-removal network, preserving the essential fault signals while removing background noise. This is evident in the frequency feature curves, where the intensities of fault frequencies are enhanced by *>*3 dB, and in the temporal feature curves, which retain the intermittent fault signals from similar background noise signals and achieve an average *>*2.5 dB reduction in background noise. To demonstrate the benefit of background removal, different classification networks were trained using both the RWD collected from the simulation test site and the corresponding background-removed data, under the same network structure and parameter settings. The results on the same test data are shown in Fig. 6b. The training dataset includes 15 samples for each class, whereas the test data includes 30 samples for each class. All networks were trained with *>*200 epochs to reach a convergent state. The results indicate that the denoised data demonstrate accuracy improvements of 8.4, 4.2, and 1.6% in CNN, ResNet, and CNN-BiLSTM, respectively, when compared to the initial data. From the statistical comparison between CNN-initial and CNN-denoised, it shows a statistically significant difference in classification accuracy between the two networks. This demonstrates that applying the noise-removal network effectively improves the final classification performance, confirming that the noise-removal network can successfully suppress noise while preserving event-related features.

Given the inherent randomness in the network training, multiple training rounds with different random parameter settings were performed. After ten rounds of training, the CNN achieved average accuracies of 74.42% for denoised data and 70% for initial data, with standard deviations of 1.71 and 3.8%, respectively. This confirms the background noise removal capability of the noise-removal net. The denoised data show performance improvements across different network architectures, especially in simpler networks. Statistical evaluation of classification results is provided in the Supplementary Materials. The performance of initial and denoised data is compared across small training datasets of varying sizes, as shown in Fig. 6c.

### Field test of belt conveyor fault detection

**Fig. 7:**
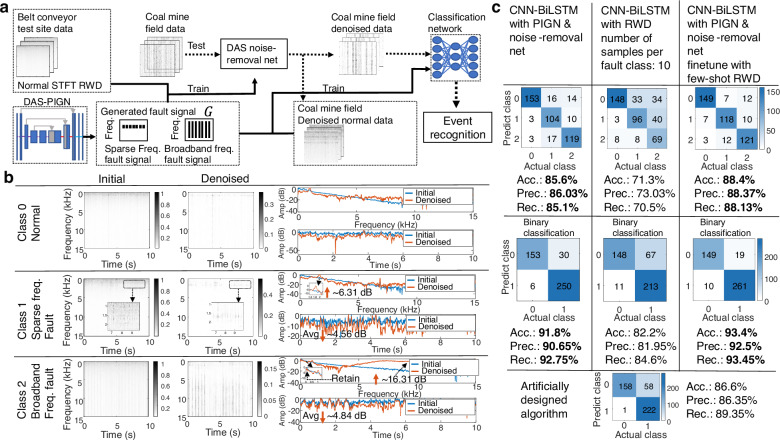
Deployment of physics-informed DAS neural network paradigm in the coal mine field. **a** Training and testing processes of PIGN, noise-removal net and classification network in the coal mine field. The noise-removal network, trained using background data from the belt conveyor simulation test site and PIGN-generated data, can be directly applied to remove background noise in field data from the coal mine. The training of the classification network does not require fault data from a simulation test or field. Dashed lines indicate the testing phase and solid lines indicate the training phase. **b** Comparison of initial and denoised data in the coal mine field of different classes. The noise-removal net, deployed directly from the belt conveyor simulation test site to the coal mine field, can still remove background noise and retain fault signals. **c** Fault recognition results of artificially designed algorithm and classification networks trained with denoised PIGN-generated data or RWD. Class 0, normal; Class 1, sparse frequency fault; Class 2, broadband frequency fault. In a binary classification task, class 0 and class 1 represent normal and fault, respectively. Physics-informed DAS neural network paradigm with PIGN and noise-removal net shows better classification performance than data-driven network and artificially designed algorithm

In the practical deployment of DAS, different sites of the same application often have distinct environments, resulting in poor generalization of traditional DAS data processing algorithms. For a specific application, events to be detected across different sites follow a similar basic physical model, and the corresponding DAS data should have similar features. The main difference in DAS data for these similar events at different sites lies in the environmental background noise. The proposed neural network paradigm, which leverages event data generated from PIGN and easily obtainable background data for training, facilitates fast deployment in different fields. Figure 7a illustrates the process of deploying a fault diagnosis network in a coal mine field of belt conveyor fault monitoring. The intrinsic mechanical vibration of the belt conveyor constitutes the primary background noise. Despite significant differences in machine type, running speed, load weight, and working environment between the simulation test site and the coal mine field, the background noise at both sites originates from mechanical rotating equipment vibrations. Hence, the noise-removal net originally trained for the simulation test site can be directly applied to the coal mine field to remove background noise. The application of the noise-removal network in the coal mine field scenario follows the same procedure as illustrated in Fig. 4f. The denoised results of different types of events are shown in Fig. 7b. The noise-removal net remains effective for field data, reducing background noise by *>*4 dB in the time feature curves, while significantly enhancing fault signal intensity by more than 6 dB in the frequency-domain feature curves. Even though the amplitude of background noise in the coal mine field is higher than that of the simulation test site, the denoising network still shows effective background noise removal capability. Normal data consisting of 450 samples collected at different times and locations over a single day at the coal mine field are processed by the noise-removal network to obtain denoised real-world normal data. Subsequently, the classification network is trained using PIGN-generated fault data and real-world background-removed normal data as inputs, corresponding to fault and normal classes, respectively. Importantly, real-world event data from the coal mine field is not involved in the training of any part of the network. During a four-month field monitoring period, a total of 23 roller faults occurred on the belt conveyor. Approximately ten samples were selected for each roller fault, combined with the normal data randomly selected at any time period during the 4 months, to form the test dataset. The performance of CNN-BiLSTM trained with generated and denoised normal data on the test dataset is shown in Fig. 7c. For comparison, the same network structure was trained with labeled RWD as training data. The real-world training dataset is an imbalanced dataset, in which 450 samples are in the normal class, and ten samples are in each of the two fault classes. Data augmentation of directly copying fault data and cost-sensitive learning methods are adopted to complete this unbalanced sample training task (see Supplementary Materials for details). The proposed approach, without requiring any real-world fault data for training, achieves a three-class classification accuracy of 85.6%, which is a 14.3% improvement compared to the 71.3% accuracy of the network trained with RWD. According to the statistical significance analysis in Fig. 7c, the network trained on PIGN-generated data exhibits a statistically significant improvement in classification performance compared with the network trained on RWD. This demonstrates the effectiveness of PIGN-generated data in serving as a surrogate for RWD in network training.

For practical applications, it is typically only necessary to establish whether a fault exists. Therefore, binary classification results are presented and additionally compared with a complex, manually designed fault diagnosis algorithm based on time-frequency analysis. This algorithm was deliberately designed after a comprehensive review of data spanning more than four months and has undergone meticulous parameter adjustment. Even with these manual efforts, the accuracy of the artificially designed algorithm is 86.6%, inferior to the accuracy of 91.8% achieved by the proposed paradigm. In practical use, once a small amount of real-world target event data were obtained, finetuning the network with RWD can further enhance fault diagnosis performance. After finetuning the pretrained network with labeled RWD consisting of ten samples per fault class, the accuracy for three-class and two-class classification reached 88.4 and 93.4%, respectively.

## Discussion

The gap between features of PIGN- generated data and RWD determines the performance of the noise-removal net and the accuracy of event recognition within the proposed paradigm. In the cases presented in this paper, the physical models for shake and walk events are based on elementary mechanics and vibration theory, while the establishment of feature functions for belt conveyor faults originates from the expertise of field workers and research summaries of fault signal characteristics with similar mechanical structures. In the two presented applications, the feature functions for the target events are simplified and do not fully consider factors such as fiber deployment or the coupling between acoustic or mechanical waves and the optical cable. Nevertheless, PIGN is still able to generate useful data for training the corresponding classification networks. This is because, although it is difficult to obtain complete and accurate physical models of target events in complex real-world scenarios, the basic characteristics of event signals can still be roughly described by simplified models or expert knowledge. The similarity of these basic characteristics across different sites is the main reason why data generated by PIGN based on physical models or expert knowledge can be effective. When applying the proposed method to complex scenarios, collaboration with domain experts is needed to obtain the basic physical models or relevant experience of the target events. The development of more accurate and comprehensive physical models depends on progress in related research fields. In scenarios where it is difficult to build physical models, one possible solution is to summarize the signal features of traditional acoustic sensor data to define the feature functions of target events, since such data have been shown to share certain similarities with DAS data^38^. It is worth noting that the two application scenarios examined in this study involve relatively simple environmental perturbations and event behaviors compared with the broader and more complex range of DAS applications.

One of the biggest obstacles in DAS applications is that the signal characteristics of events vary across different applications and sites, which means that DAS data processing algorithms are usually limited to solving specific tasks at particular sites. The root cause is the diversity of background noise and event signals at different work sites. In the proposed network paradigm, by employing a noise-removal network and fully leveraging easily available background data, background noise signals can be effectively eliminated. Physical models or expert knowledge can typically represent the basic shared features or significant distinctions of the same type of event across different sites. Due to the shared characteristics of the events, the physics-informed DAS neural network can be directly deployed at different sites for event identification. However, PIGN-generated data is struggle to capture the specific signal differences of events at individual sites with the general and simplified physical model. By adopting pretraining techniques, networks trained on generated data for general scenarios can further learn the subtle, site-specific signal features from RWD for better performance.

The proposed network paradigm primarily focuses on the incorporation of physical information and training methods. In this article, three different classification network architectures are evaluated to demonstrate that the proposed paradigm can enhance classification performance across various network structures. Given the varying performance of different network architectures on different types of data, appropriate classification networks can be selected based on the characteristics of the data in practical DAS applications. The proposed paradigm also includes a data generation network and a denoising network, both of which are based on the U-Net architecture in this study. In theory, since the paradigm does not modify the specific architecture of the networks, it can be applied to alternative data generation and denoising network structures. Optimizing the architectures of the data generation, denoising, and classification networks based on the characteristics of DAS data in different applications can lead to improved results.

In conclusion, the proposed physics-informed DAS neural network paradigm can generate massive DAS target event data according to physical information and has the ability to remove intense background noise. PIGN is validated in two different DAS applications to generate different types of DAS event data. Classification networks are trained using generated DAS spatiotemporal data of shake and walk events and tested on a public dataset, which verifies the effectiveness of the data generated by PIGN. For the belt conveyor monitoring application with intense background noise, fault DAS time-frequency data generated by PIGN are used to train the DAS noise-removal net. In the belt conveyor simulation test site, the classification accuracy of denoised data showed an 8.4% improvement compared to the initial data with the CNN network. Results from transferring the networks from the simulation test site to the coal mine field demonstrate the proposed paradigm’s rapid deployment capability across different sites of the same application without requiring any real-world target event data, as well as superior event recognition performance compared to data-driven neural networks and carefully designed conventional algorithms. After learning the physical characteristics of DAS event data, the network demonstrates improved generalization and adaptability to different application sites. Further incorporating small batches of RWD for finetuning can enhance event recognition performance in specific application settings. The two presented application scenarios correspond to training based on physical models and experiential knowledge, respectively, demonstrate the preliminary effectiveness of our approach across DAS applications with varying degrees of model availability. The proposed paradigm offers a physics-informed neural-network method for signal processing in optical sensing, imaging, and communication, providing a potential direction for mitigating long-standing challenges associated with reliance on real-world target event data and susceptibility to strong environmental noise.

## Materials and methods

The details of all content in Methods, as well as more results, are provided in the Supplementary document.

### Experiments

The DAS system, ixDAS-4000, used for belt conveyor monitoring, is provided by Ningbo AllianStream Photonics Technology Co., Ltd. The working principle of ixDAS-4000 is based on time-gated digital optical frequency domain reflectometry (TGD-OFDR). In the belt conveyor monitoring experiments, the DAS interrogator collects differential phase data. The maximum sensing range is 60 km. The minimum spatial resolution is 3.6 m. The self-noise level at 1 km is 10 $p\varepsilon \,{\sqrt{{Hz}}}^{-1}$@20 Hz and 5 $p\varepsilon \,{\sqrt{{Hz}}}^{-1}$ @100 Hz. When applying DAS to belt conveyor monitoring, it is necessary to lay the optical cable along the frame of the belt conveyor to ensure that the optical cable is in contact with the frame of the belt conveyor. When a roller malfunctions, the acoustic waves generated by the fault propagate through the frame and couple into the optical fiber, allowing the DAS system to detect them.

The simulation test site for the belt conveyor is provided by Ningbo AllianStream Photonics Technology Co., Ltd. The total length of the belt conveyor at the simulation test site is 13 m. The roller spacing is 1.2 m. The diameter of the roller is 159 mm. The belt speed of the conveyor can be adjusted between 0 and 5 m/s. The belt conveyor is in an unloaded state during operation. Three kinds of roller faults were manufactured manually, including eccentric, split and cracked roller. The fiber length is about 250 m.

The total length of the belt conveyor at the coal mine field is about 400 m. The fiber length is about 1 km. The roller spacing is ~1.2 m. There are two types of belt conveyor roller diameters, 133 and 159 mm. The conveyor operates under loaded conditions, and data were collected over a period of more than 4 months. During operation, the ambient noise level at the site is high, measured at ~100 dBA using a sound level meter (Model AWA 5653). For belt conveyor experiments, the data- acquisition interval exceeds 2 min, ensuring that collected samples are independent and eliminating concerns about data leakage.

For the public dataset, two different fiber lengths were used: 5 and 10 km. The training and testing data were randomly split. Since the partition strategy is not clear and detailed, the classification results obtained from networks trained on a public dataset may be subject to potential data leakage.

### Physical model

The selection of the physical-model parameters did not rely on any information from the test data. Overall, the parameters were chosen to span a relatively broad range for each event so as to capture potential signal variations across different scenarios.

### Physical model of DAS

When the external perturbations are coupled to the optical fiber, stress is applied to the optical fiber to change its length, resulting in the phase change of light propagation on the optical fiber. The phase change of the Rayleigh backscattered light field in the fiber detected by DAS corresponds to changes in external strain or acoustic waves. Therefore, the physical model of target events in DAS applications should reflect the force acting on the optical fiber or the changes in its length (see Supplementary Material for details). We introduce a scaling factor M that accounts for the difference between the physical quantities in the model (e.g., force or displacement) and the real DAS data. Although the initial modeling considers only the force or length change caused by the event, the application of the scaling factor provides an approximate mapping to the corresponding DAS signal (for example, phase response).

### Shake event

Shake can be regarded as the combination of forced vibration caused by external force and free vibration of optical fiber. The position of a point on the optical fiber *x*(*t*) can be expressed as:

5 $$x(t)={A}_{1}{e}^{-\delta t}\,\cos (\Omega t+\psi )+B{F}_{0}\,\sin (\omega t-\phi )$$

where *A*_1_, *δ*, Ω and *ψ* are the initial amplitude, attenuation coefficient, vibration frequency and initial phase of free vibration; *B*, *ω* and *ϕ* are coefficient, vibration frequency and initial phase of forced vibration; *F*_0_ is the initial amplitude of external force. The change of fiber position can reflect the change of fiber length, which is equivalent to the change of DAS signal. A scaling factor *M* (*t*) is used to approximate the process of fiber position change to the DAS signal. Therefore, the temporal feature function *T* of shake event can be expressed as

6 $$T=M(t)x(t)+N(t)$$

where *N* is the random noise primarily accounts for noise from the photodetector and various unquantifiable environmental disturbances. The projection of shake data in space has no obvious features, so the spatial feature function *S* can be expressed as

7 $$S=rand(s)+N(s)$$

where *s* is the spatial channel. Because a shake event is easy to make, the signals on adjacent spatial channels of DAS have correlation; it is necessary to properly consider the spatial-correlation constraint function when training PIGN for shake data generation. The detailed definitions, numerical ranges and selection criteria of the above parameters are in the Supplementary Materials.

### Walk event

Since the walking speed of pedestrians follows a sine function, the acceleration *a*(*t*) of pedestrians follows a cosine function, and can be expressed as:

8 $$a=\frac{\pi {v}_{c}}{c}\,\cos \left(\frac{\pi t}{c}\right)$$

where *v*_*c*_ is the amplitude of speed change, *c* is angular velocity coefficient. For different walking modes, such as fast walking and slow walking, the values of *v*_*c*_ and *c* are different. According to Newton’s second law of motion, the force exerted by pedestrians on the ground can also be approximately considered as a cosine variation and acts on the optical fiber laid on the ground. Therefore, the temporal feature function *T* of a walk event can be expressed as:

9 $$T={M}_{fast}(t){a}_{fast}+{M}_{slow}(t){a}_{slow}+N(t)$$

where *M* is the scaling factor. The function includes both fast and slow walking modes. The spatial feature function modeling of the walk event is relatively complex, and here we simply approximate it with random numbers:

10 $$S=rand(s)+N(s)$$

Supplementary Materials include the detailed definitions, numerical ranges, and criteria used for selecting the above parameters.

Noted that the total duration of 1000 time samples of the public dataset is estimated to be roughly 10 s, since it does not provide accurate information on the repetition frequency, as detailed in the Supplementary materials. The lack of an exact repetition frequency has only a minor impact on our method, since both the RWD and the PIGN-generated data are inherently constrained within physically plausible parameter ranges and share similar characteristic features. As a result, the PIGN-generated data remain effective for training the classifier, as reflected in the recognition performance shown in Fig. 3c.

### Belt conveyor fault event

The faults in belt conveyors are mostly caused by rotating components such as rollers, so the temporal feature function of the fault signal can be approximately expressed as:

11 $$T=M(t)\sin \left(\frac{2\pi }{P}t+\varphi \right)+N(t)$$

where *M* is the scaling factor, *P* is the rotational period of the roller. In belt conveyor fault detection, diagnosis relies on analyzing the time-frequency characteristics of DAS signals at fixed spatial positions, rather than using the spatiotemporal data directly. Therefore, in this case, only the temporal feature function and frequency domain feature function will be established to guide the training of PIGN. According to the reference on fault analysis of rotating machinery equipment and the experience of on-site workers^56^, the frequency feature of fault signals can be mainly divided into two classes: sparse frequency and broadband frequency. To simplify the model, the single frequency of the fault signal in the frequency domain is approximated by the probability density curve of a Gaussian distribution. Sparse frequency feature function can be expressed as:

12 $$F(f)=\mathop{\sum }\limits_{i=1}^{N}{A}_{i}\frac{1}{{\sigma }_{i}\sqrt{2\pi }}{e}^{-\frac{{(f-{\mu }_{i})}^{2}}{2{{\sigma }_{i}}^{2}}}+N(f)$$

where *A*_*i*_, *µ*_*i*_ and *σ*_*i*_ are the amplitude, center frequency and bandwidth of the *i*th fault frequency, respectively. Broadband frequency feature function can be expressed as:

13 $$F(f)=rand(f)+N(f)+{A}_{0}$$

where *A*_0_ is the basic frequency amplitude value to ensure that the signal has signal at all frequencies for simulation of broadband signal. The Supplementary Materials contain comprehensive information on the definitions, ranges, and selection rules for the above parameters. Importantly, we did not use any specific information from the coal mine site to tune the PIGN network.

### PIGN structure

PIGN is based on the U-net structure^58^. There are two main types of building blocks in PIGN: convolution block(3 × 3 convolution layer + Leaky ReLU + MaxPool) and up-convolution block (2 × 2 transpose convolution layer + Leaky ReLU). The input and output data size of PIGN for generation of shake and walk data were 1000 × 12, while the size for belt conveyor fault generated data is 1025 × 584. All the networks in this article are implemented on the Pytorch platform, and a NVIDIA GeForce RTX 4090 is used for training.

In untrained mode for training, PIGN only inputs a single random image to output an individual event data with only a single set of feature curves as its training target. Once training is complete, the PIGN can generate the corresponding DAS data. Therefore, in untrained mode, PIGN is more suitable for generating a small number of data or data with specific features. The whole training time with 10,000 epochs for the generation of an individual event data is about 40 s in PIGN to generate shake and walk events, while it takes about 95 s with 15,000 epochs to generate an individual belt conveyor fault time-frequency data.

### Noise-removal net structure

DAS noise-removal net is based on a U-net structure. The network layers of the noise-removal net are the same as PIGN. In the application of belt conveyor monitoring, 3960 samples of data with a size of 1025 × 584, which were composed of 100 normal background data from the belt conveyor simulation test site combined with PIGN-generated fault data, are used as input data for the noise-removal net training process, label data is the corresponding background data. The training process of the network reached convergence after 150 epochs, taking a total time of ~3 h. During the testing process, it only took about 0.003 s to simultaneously remove background noise from eight samples of data.

### Classification networks structure

Three types of networks are employed as classification networks, namely CNN, ResNet, and CNN-BiLSTM. These three types of neural networks are classic representative network structures in the development of neural networks. Most network structures are based on these three types of networks. The CNN network is the basis of most current network structures. The CNN network adopted in this article consists of two convolutional layers and one fully connected layer. ResNet is the representative of a deep network. The standard ResNet34 is adopted in the article. CNN-BiLSTM, a classic combination of convolutional and recurrent layers, can extract both local and global features from data, making it well-suited for spatiotemporal signal processing. The CNN-BiLSTM model used here contains three convolutional layers, one BiLSTM layer, and one fully connected layer. These networks generally reach convergence after training 100 epochs.

## Supplementary information

Supplementary materials

## Data Availability

The data that support the findings of this study are available from the corresponding author upon reasonable request.

## Code availability

The data that support the findings of this study are available from the corresponding author upon reasonable request.
