# Supplementary material for: Towards a physics-informed network paradigm with data generation and background noise removal for different distributed acoustic sensing applications
Source: Light Sci Appl. 2026 Jun 23;15:281. doi: 10.1038/s41377-026-02295-5 (PMC13291359; doi:10.1038/s41377-026-02295-5)
Supplement: Supplementary file 1 — Supplementary materials [file 41377_2026_2295_MOESM1_ESM.docx]

**Supplementary Information for: Towards a physics-informed network paradigm with data generation and background noise removal for different distributed acoustic sensing applications**

YANGYANG WAN ^1^, HAOTIAN WANG^1^, XUHUI YU^1^, JIAGENG CHEN^1^, XINYU FAN^1^, AND ZUYUAN HE^1, *^
1 State Key Laboratory of Photonics and Communications, Shanghai Jiao Tong University, 800 Dongchuan Road, Minhang, Shanghai 200240, China

* Corresponding author: zuyuanhe@sjtu.edu.cn

**Contents**

[1. Physical model 2](#_Toc225259146)

[1.1 Physical modeling of DAS signal 2](#_Toc225259147)

[1.2 Physical modeling of shake 3](#_Toc225259148)

[1.3 Physical modeling of walk 6](#_Toc225259149)

[1.4 Physical modeling of belt conveyor 8](#_Toc225259150)

[1.5 Real-world constraint function 11](#_Toc225259151)

[1.6 DAS constraint function 14](#_Toc225259152)

[2. Physics-informed generative network, PIGN 15](#_Toc225259153)

[2.1 Full loss function 15](#_Toc225259154)

[2.2 The training mode of PIGN 16](#_Toc225259155)

[2.3 PIGN structure 16](#_Toc225259156)

[2.4 Generated shake and walk data by PIGN vs real-world data 19](#_Toc225259157)

[2.5 Generated belt conveyor fault data by PIGN vs real-world data 22](#_Toc225259158)

[3. Noise-removal net 27](#_Toc225259159)

[3.1 Principle and Structure of noise-removal net 27](#_Toc225259160)

[3.2 Denoised results 30](#_Toc225259161)

[4. Classification networks 34](#_Toc225259162)

[4.1 Structures of classification networks 34](#_Toc225259163)

[4.2 Supplementary data of classification networks 49](#_Toc225259164)

[5. Statistical significance tests 51](#_Toc225259165)

[5.1 Statistical test methods 51](#_Toc225259166)

[5.2 Statistical significance tests results 52](#_Toc225259167)

[6. Experiment 61](#_Toc225259168)

[6.1 Experimental details of public datasets 61](#_Toc225259169)

[6.2 DAS system in the belt conveyor fault monitoring 62](#_Toc225259170)

[6.3 Sites of belt conveyor 63](#_Toc225259171)

[6.4 Experimental details of belt conveyor fault monitoring 64](#_Toc225259172)

[7. Supplementary data and enlarged figures related to the main text 68](#_Toc225259173)

[Reference 70](#_Toc225259174)

# 1. Physical model

## 1.1 Physical modeling of DAS signal

Figure S1 Schematic diagram of optical fiber affected by external force

When the external force acts on the optical fiber, the length of the optical fiber will change according to the elastic deformation theory[1], which can be expressed as

$\Delta F=-k\Delta L$ (1)

where $k$ is elastic coefficient of optical fiber. The strain on the optical fiber is $\varepsilon=\frac{\Delta L}{L}$. According to the optical fiber sensing theory, the strain on the optical fiber will change the distribution of the optical fiber refractive index[2], which can be expressed as

$\Delta n=-\frac{1}{2}n^{3}p_{e}\varepsilon=C_{\varepsilon}\varepsilon$ (2)

where $\Delta n$ is the change in refractive index, $p_{e}$ is stress-optical coefficient. DAS signal is differential phase, which can be expressed as

$\Delta\phi_{L}=\phi_{B}-\phi_{A}=2\beta(n+C_{\varepsilon})\varepsilon L=2\beta(n+C_{\varepsilon})\Delta L=2\beta(n+C_{\varepsilon})\frac{-\Delta F}{k}$ (3)

where $\beta$ is propagation constant. From Equation (3), the DAS signal has a linear relationship with external force or change in fiber length. Therefore, the modeling of external events should ultimately focus on the force acting on the optical fiber or change in the length of the fiber.

**The definition for the scaling factor M(t).**

Since our simple physical models are constructed based on the analysis of forces or displacements during shake, walk, and roller fault events, there is inevitably a gap between the modeled signals and the actual strain signals measured by the optical fiber. To address this discrepancy, we introduce a scaling factor M that accounts for the difference between the physical quantities in the model (e.g., force or displacement) and the real DAS data. As the physical models are simplified and often produce regular patterns, they cannot fully capture the complexities of real-world events. Therefore, we allow the scaling factor M(t) to vary over time, using different functional forms depending on the specific event, in order to better approximate the temporal variability observed in actual DAS data.

## 1.2 Physical modeling of shake

Figure S2 Schematic diagram of modeling shake event on optical fiber

The optical fiber under shake event can be simplified into forced vibration and free vibration[3], as shown in Figure S2. External applied force can be expressed as

$f(t)=F_{0}\cos(\omega t)$ (4)

where $F_{0}$ is the initial amplitude of external force, and $\omega$ is the frequency of force. With these two types of vibrations, the position of point A on the optical fiber can be expressed as

$x(t)=A_{1}e^{-\delta t}\cos(\Omega t+\psi)+BF_{0}\sin(\omega t-\phi)$ (5)

where $A_{1}$, $\delta$, $\Omega$ and $\psi$ are the initial amplitude, attenuation coefficient, vibration frequency and initial phase of free vibration; $B$and $\phi$ are coefficient and initial phase of forced vibration. For the convenience of analysis, the position of point A $x(t)$ can be approximated as the change in fiber length. Therefore, the time feature function of the shake event on DAS data can be expressed as

$T=M(t)x(t)+N(t)$ (6)

where $M$ is the scaling factor, and $N$ is the noise, including random noise, DAS phase noise and fading noise. For the shake event, the scaling factor is modeled as a time-varying random variable uniformly distributed between 1 and 3. This design reflects the fact that, in real-world shaking processes, the applied force is not constant over time. Therefore, introducing randomness into the scaling factor helps to simulate this variability. The lower bound of 1 is chosen because, during the shake event, there is generally a consistent presence of force throughout the process. We assume that the change in applied force will be at least twice the minimum value, therefore we randomly selected 3, an integer larger than 2, as the upper bound.

The spatial feature function of shake can be expressed as

$S=rand(s)+N(s)$ (7)

where $s$ is the spatial channel. The spatial characteristics of the corresponding events are either not obvious or difficult to model, so we use random functions to represent the spatial features. In this work, noise functions are employed for this purpose.

Accordingly, our framework needs feature functions in two dimensions as targets for generating 2D DAS data, as stated in the previous manuscript. Because the spatial behaviors of the shake and walk events are difficult to model analytically, we use a noise function to approximate their spatial feature functions. This serves two purposes: (1) it supplies the PIGN with the necessary orthogonal projection curves to guide the generation of 2D image data, and (2) the randomness of the noise function can approximate the diverse and complex spatial patterns that these events may exhibit.

**The definition for the noise component *N(t)*.**

The random noise primarily accounts for shot noise from the photodetector and various unquantifiable environmental disturbances. This is modeled using additive Gaussian white noise for simplicity.

The phase noise in DAS is mainly attributed to the phase noise of the laser source. In general, narrower laser linewidths and shorter sensing distances lead to lower phase noise. In typical short-pulse DAS systems, phase noise is usually negligible compared to environmental disturbances.

Fading noise arises from the coherent detection scheme used in DAS systems. Due to polarization mismatch between the backscattered Rayleigh signal and the local lightwave, as well as destructive interference effects, the optical intensity at certain fiber locations can become very low. This results in erroneous phase demodulation, often manifesting as ±π phase jumps in the phase data of DAS. The occurrence and severity of fading noise depend on the specific DAS system used. In our experiments, the DAS device is equipped with fading noise mitigation techniques, so its influence can be largely ignored. In the public dataset we used, no obvious fading noise was observed, and its impact is also considered negligible.

For the shake event, the noise component is modeled using Gaussian white noise with a mean of 0 and a standard deviation of 0.3. Because the SNR of real-world data cannot be known in advance, we can only use the standard deviation of the noise to approximate its typical fluctuation range. Since DAS data represent phase information, the actual signal amplitude does not exceed π. In our setting, we assume that the noise amplitude is roughly one-tenth of the signal amplitude. Therefore, we set the standard deviation to 0.3 (approximately π/10).

**Other** **related parameters of the Shake event feature curve functions**

For the free vibration component, the initial amplitude is randomly selected between 0.1 and 0.6. Because the intensity of free vibration in the shake event is much lower than that of forced vibration, we set its amplitude upper bound to 0.6. Although the amplitude of free vibration is small, it is still present; therefore, we set its lower bound to 0.1. The attenuation coefficient is set to zero and thus not reflected in the generated feature curves. The vibration frequency is chosen randomly between 0.5 Hz and 5 Hz to represent the typical low-frequency behavior of free vibration. The initial phase is a random value between 0 and 2π.

For the forced vibration component, the product of the coefficient and the initial amplitude of the external force is treated as a random value between 1 and π. For forced vibration, the shaking action persists, so we set the amplitude lower bound to 1. To account for the possibility of strong shaking, we set the amplitude upper bound to the maximum value of π. The vibration frequency is randomly selected from 60 Hz to 350 Hz, corresponding to the typical high-frequency oscillations observed in tensioned cables. The initial phase is also a random value between 0 and 2π.

For the spatial feature function, we define it as a random variable that varies along the spatial (channel) dimension, with values ranging from 0 to π (due to the limitation that DAS signals are phase information).

For the shake event, the vibration frequencies for the free-vibration and forced-vibration components were selected as 0.5 – 5 Hz and 60 – 350 Hz, respectively. The reason is as follows:

According to the Reference ([Sound - Standing Waves, Frequency, Wavelength | Britannica](https://www.britannica.com/science/sound-physics/Standing-waves?utm_source=chatgpt.com), equation(22)), the achievable vibration frequency $\boldsymbol{f}$ of a rope depends on its tension $\boldsymbol{F}$, length $\boldsymbol{L}$, and linear density $\boldsymbol{\mu}$:

$$\boldsymbol{f}\mathbf{=}\frac{\sqrt{\boldsymbol{F}\mathbf{/}\boldsymbol{\mu}}}{\mathbf{2}\boldsymbol{L}}$$

In the public dataset, the fiber was described as being fixed to an iron cage, but the number of fixation points and the distances between them were not specified. Moreover, the linear density $\boldsymbol{\mu}$ of the fiber was not available. Therefore, we referred to typical linear densities of conventional ropes (0.01–0.2 $\mathbf{kg}\mathbf{m}^{\mathbf{-1}}$) to estimate the possible frequency ranges.

For short, highly tensioned, and lightweight rope segments (e.g., length of 1 m, tension of 1000 N and linear density of 0.01 $\mathbf{kg}\mathbf{m}^{\mathbf{-1}}$ will achieve a fundamental frequency of ~158 Hz), the fundamental frequency can reach tens to hundreds of hertz, with higher harmonics being integer multiples. In contrast, for long, loose, or heavier ropes (e.g., length of 20 m, tension of 80 N and linear density of 0.2 $\mathbf{kg}\mathbf{m}^{\mathbf{-1}}$ will achieve a fundamental frequency of ~0.5 Hz), the fundamental frequency may fall within the range of approximately 0.5–5 Hz.

Based on these considerations, we selected 0.5–5 Hz as the frequency range representing free vibration of the fiber, and 60–350 Hz as the range representing the forced-vibration frequencies that may occur in shorter, tensioned segments of the fiber.

## 1.3 Physical modeling of walk

Figure S3 Schematic diagram of modeling walk event on optical fiber

Related research has shown that the walking speed of pedestrians can be expressed as[4]

$v=v_{0}+v_{c}\sin(\frac{\pi t}{c})$ (8)

where $v_{0}$ is the average speed, $v_{c}$ is the amplitude of speed change, and c is angular velocity coefficient. High c value corresponds to slow walking, and low c value corresponds to fast walking. Hence, the acceleration $a$ of pedestrians walking can be expressed as

$a=\frac{\pi v_{c}}{c}\cos(\frac{\pi t}{c})$ (9)

According to Newton's second law of motion, the force exerted by pedestrians on the ground during walking is proportional to the walking acceleration. Because the force will eventually be transmitted to the optical fiber with a certain attenuation, the temporal feature function of DAS signal for walking events can be approximately expressed as

$T=M_{fast}(t)a_{fast}+M_{slow}(t)a_{slow}+N(t)$ (10)

The spatial feature function of walk can be expressed as

$S=rand(s)+N(s)$ (11)

The spatial characteristics of the corresponding events are either not obvious or difficult to model, so we use random functions to represent the spatial features. In this work, noise functions are employed for this purpose.

**Related parameters of the walk event feature curve functions**

For the walk event, both fast walking and slow walking are considered, their scaling factors are modeled as a time-varying random variable uniformly distributed between 0.5 and π. This is intended to capture the variability in footstep force over time. The choice of 0.5 as the lower bound is based on the fact that the walk event is a continuous activity and thus always present; we therefore selected a random value greater than 0 but less than 1 to simulate occasional low-amplitude situations. The upper bound is set to the maximum value of π to account for cases where walking can sometimes generate relatively strong signals. As with the shake event, the noise component in the walk scenario is also modeled using additive Gaussian white noise, with a mean of 0 and a standard deviation of 0.3 (The reason for choosing 0.3 is the same as above in the shake event).

In the walk event, the amplitude of speed change serves as the amplitude term in the temporal feature function and is ultimately multiplied by a time-varying scaling factor. Since the scaling factor is a random variable, we did not explicitly specify the amplitude of speed change itself. The angular velocity coefficient reflects the walking speed: for slow walking, it is randomly set between 1 and 0.333, corresponding to a typical slow walking frequency range of approximately 0.5 Hz to 1.5 Hz. For fast walking, the coefficient is randomly set between 0.333 and 0.166, corresponding to a faster walking frequency range of approximately 1.5 Hz to 3 Hz. Regarding the spatial feature function, it is defined as a random variable that varies with the spatial (channel) position, with values ranging from 0 to π (The reason for choosing the parameter range is the same as above in the shake event).

For the walk event, the angular velocity coefficient was set to 1–0.333 for slow walking and 0.333–0.166 for fast walking, corresponding to walking frequencies of 0.5–1.5 Hz and 1.5–3 Hz, respectively. The reason for these parameter ranges is based on Reference (Tudor-Locke, C., Ducharme, S. W., Aguiar, E. J., Schuna Jr, J. M., Barreira, T. V., Moore, C. C., ... & Staudenmayer, J. (2020). Walking cadence ($\mathbf{steps}\mathbf{min}^{\mathbf{-1}}$) and intensity in 41 to 60-year-old adults: the CADENCE-adults study. International Journal of Behavioral Nutrition and Physical Activity, 17(1), 137.) reporting that younger adults typically take around 100 steps per minute (approximately 1.66 Hz), which is widely considered the threshold for moderate-intensity walking. The study indicates that adult walking frequencies generally range from approximately 40 steps per minute (~0.66 Hz) to 160 steps per minute (~2.66 Hz), as shown in Fig.2 of the Reference. To cover a broader range, we set the slow-walking frequency range to 0.5–1.5 Hz and the fast-walking range to 1.5 – 3 Hz.

## 1.4 Physical modeling of belt conveyor

Figure S4 (a) Schematic diagram of belt conveyor. (b) Expert experience of belt conveyor fault in frequency domain.

As stated in Ref.[8]:“Acoustic and vibration data from rotating machinery have long been used for fault detection. These data usually contain both narrowband signals as well as broadband random components, which in combination effectively mask the fault-induced impulsive signals.”, rotating machinery typically exhibits both narrowband and broadband frequency components. This implies that, when a fault occurs, changes will manifest in both types of components. Combined with field workers’ feedback:“ some faulty rollers produce sharp, piercing tones (corresponding to a dominant sparse frequency), while others simply sound louder overall (corresponding to enhanced broadband components) ”, we categorized belt conveyor roller faults into sparse-frequency and broadband-frequency faults.

Ref. [7] (Fig. 1) confirms that different types of faults give rise to distinct frequency characteristics, such as a single prominent harmonic or multiple harmonic orders. Ref. [9] (Fig. 2) shows that, under ideal conditions, rotating machinery fault signals exhibit periodic impulsive structures in the time domain. Based on these studies, together with the feedback from field workers, we defined the temporal feature function in our model using a sinusoidal form, and we divided the frequency feature function into sparse-frequency and broadband-frequency categories.

The basic and key structure of belt conveyor is rotary roller, which be simplified as a rotor. Fault rotating equipment usually generates fault signals periodically in time due to its rotating characteristics. According to the diameter $D$ of the rotor and the speed $v$ of belt transmission, the period $P$ of fault signal generation can be approximately expressed as $P=\frac{D\pi}{v}$. Therefore, the temporal feature function of belt conveyor fault can be expressed as

$T=M(t)\sin(\frac{2\pi}{P}t+\varphi)+N(t)$ (12)

Theoretical modeling of belt conveyor roller faults is relatively rare, and the modeling differs greatly from the actual situation. Therefore, expert experience is used here to construct the frequency feature function of the fault. Based on the summary of relevant researches and the experience of on-site staff[5], it can be roughly divided into two classes in the frequency domain: sparse frequency and broadband frequency for the faulty roller of the belt conveyor, as shown in Figure S4(b). Faults with sparse frequency feature are usually rotor misalignment, rotor eccentricity and shaft bending. Faults with broadband frequency feature are usually rotor friction and cavitation. The sparse frequency feature function can be expressed as

$F(f)=\sum_{i=1}^{N} A_{i}\frac{1}{\sigma_{i}\sqrt{2\pi}}e^{-\frac{(f-\mu_{i})^{2}}{2{\sigma_{i}}^{2}}}+N(f)$ (13)

where $A_{i}$, $\mu_{i}$ and $\sigma_{i}$ are the amplitude, center frequency and bandwidth of the *i*th fault frequency, respectively. The broadband frequency feature function can be approximately expressed as

$F(f)=rand(f)+N(f)+A_{0}$ (14)

where $A_{0}$ is the basic frequency amplitude value to ensure that the signal has signal at all frequencies for simulation of broadband signal.

The main difference between sparse frequency and broadband frequency faults is that sparse frequency faults have clear strong components at certain frequencies but low intensity in other bands, while broadband frequency faults have no obvious strong components and show a relatively uniform intensity across the entire frequency range.

**Related parameters of the belt conveyor fault feature curve functions**

In the DAS time-frequency data of the belt conveyor scenario, the generated data is limited to between 0 and 1. The reasons are as follows: (1) the amplitude ranges of data collected across different sites can vary significantly; and (2) for fault identification, the relative changes in the data are typically more important than the absolute values. Therefore, for each belt-conveyor application, all real-world data were individually normalized to the 0–1 range.

For the belt conveyor fault, the scaling factor is modeled as a time-varying random variable uniformly distributed between 0 and 1. The lower bound is set to 0 because a faulty roller does not continuously generate fault-induced acoustic signals. The upper bound is set to 1, as we do not consider any mechanisms that would further amplify the fault signal. In addition, on top of the above settings, the scaling factor is randomly set to zero within several time intervals. The duration of each zero-valued interval is randomly selected between 0.1 and 5 seconds, and the number of such intervals is randomly chosen from 0 to 5. This design is motivated by feedback from field personnel of simulation test site and historical fault audio data, which indicate that fault-induced acoustic signals do not always occur continuously. The intensity of fault sound can vary significantly and may even disappear temporarily. The lower bound of 0.1 s for the zero-valued interval was chosen as a small timescale to reflect the possibility of brief intermittent disappearances of fault-related features. The upper bound of 5 s was selected in consideration of the total duration of the PIGN-generated fault data (10 s). Setting the limit to 5 s ensures that the generated samples still retain sufficient fault-related information.

The noise in the conveyor belt working environment is highly complex and influenced by multiple uncontrollable factors. Therefore, we adopt a simplified modeling approach by introducing Gaussian white noise with a mean of 0 and a standard deviation of 0.05. Considering that the maximum signal amplitude is 1, and using a typical signal-to-noise ratio of approximately 13 dB as a reference, we set the noise standard deviation to 0.05 (i.e., 1/20).

For the rotational period of the roller P used in the temporal feature function, we considered typical roller diameters (ranging from 100 mm to 200 mm) and common belt speeds (ranging from 2 ${m s}^{-1}$ to 5 ${m s}^{-1}$) in conveyor systems. Based on these values, the resulting rotational period falls within the range of approximately 0.06 to 0.3 seconds. The phase term φ in the sine function of the temporal feature function is set as a random variable uniformly distributed between 0 and 2𝜋. This randomness is introduced to simulate the variability in the initial phase of real-world signals.

For the sparse frequency feature function, the number of sparse frequency components is randomly selected between 1 and 5. The maximum number of sparse frequencies, 5, was chosen based on feedback from on-site staff from simulation test site. The amplitude A of each component is a random value between 0.3 and 1 (The maximum signal value is 1, so the upper limit is 1; however, under sparse frequency fault characteristics, the fault frequency must have a certain intensity, so the lower limit is chosen to be 0.3), the center frequency μ is randomly selected between 1000 Hz and 8000 Hz (Since most environmental noise in the belt conveyor operating area is below 1kHz, the lower limit of the fault frequency is set at 1000Hz; Since high-frequency sound waves generally have difficulty affecting optical fibers through air coupling, sound waves exceeding 8000 Hz are difficult to detect effectively in actual DAS detection, so the upper limit is set at 8000 Hz), and the bandwidth σ is randomly selected between 10 Hz and 200 Hz (This is a randomly set parameter range. Considering that the fault frequency bandwidth should be between tens of Hz and hundreds of Hz, 10 Hz and 200 Hz were randomly selected as the lower and upper limits, respectively.). The selection of the center frequency range is based on the experience described by field workers from simulation test site. The additive noise term N(f) is similarly modeled as Gaussian white noise with a standard deviation of 0.008~0.018. The chosen range for the additive noise term’s standard deviation reflects our intention to simulate varying noise levels, as environmental noise amplitudes fluctuate in practice. The lower bound of 0.008 is selected because, according to feedback from field personnel of simulation test site and historical fault audio data, signal amplitudes exceeding 0.01 often indicate a potential fault. We therefore chose a value slightly below 0.01 to represent situations with relatively low noise. To simulate higher-noise conditions, we set the upper bound to 0.018, approximately twice the 0.01 amplitude level. This setting is applied consistently in both the sparse frequency and broadband frequency feature functions.

For the broadband frequency feature function, the basic frequency amplitude value A_0 is set to 0.01. The choice of the basic frequency amplitude value is based on feedback from field personnel and historical fault audio data, which indicate that amplitudes exceeding 0.01 of the overall spectrum often suggest the possibility of presence of a fault. The fluctuation component, rand(f), is modeled as Gaussian noise with a variance of 0.03. Because sparse fault frequencies are typically much stronger than broadband fault components, we assume that the broadband features have intensities roughly half of the minimum sparse fault frequency. Since the minimum sparse fault amplitude is set to 0.3, we therefore assign a variance of 0.03 to the fluctuation component, corresponding to most values falling below approximately 0.17.

## 1.5 Real-world constraint function

Figure S5 Example generated walk data without (a) and with (b) using the continuous signal constraint function.

To narrow the gap between generated data and RWD, constraint functions based on real-world characteristics have been incorporated into the loss function. The design of the real-world constraint function must be tailored to the specifics of the target application.

The signals generated in the real world are generally continuous signals. When using generative network to generate DAS data, due to the random output characteristics of neural network, there will be a large number of jumps in the output data, which does not conform to the characteristics of real-world data. So we propose a continuous signal constraint function to add to the full loss function of the PIGN. The discrete formulation of the constraint function in the time dimension can be expressed as

$\frac{1}{\text{n-}1}\sum_{i=1}^{n-1} (\Delta I_{i})^{2}$ (15)

The introduction of square in Equation (15) is for differentiability and convexity in network training process. Figure S5 shows the walk data generated by PIGN with and without the continuous signal constraint function. Figures S5a and S5b respectively illustrate the PIGN-generated walk data before and after the inclusion of the continuous signal constraint function. Based on our simplified walk model, the ideal signal generated by a walk event is expected to follow a sinusoidal form. However, the data shown in Figure S5a exhibit abrupt and irregular changes in the time domain, which deviate significantly from both the ideal walk signal and the typical smooth patterns observed in real-world walking scenarios. In contrast, after introducing the continuous signal constraint function, the PIGN-generated data in Figure S5b become noticeably more continuous and smoother, showing much better agreement with both the theoretical walk model and actual observed data. Without the continuous signal constraint function, the generated event data exhibit many rapid fluctuations, as shown in Fig. S5a, which contradicts the generally continuous nature of real-world signals in the time domain. With the continuous signal constraint function applied, the PIGN-generated data become smoother, and more in line with the actual situation compared with Fig. S5a.

Based on actual conditions, similar continuous signal constraint function can be constructed in the frequency domain and spatial domain.

Figure S6 Example generated shake data without (a) and with (b) using the correlation constraint function.

In some scenarios, real-world data show correlations in the spatial domain or other domain, such as time, frequency. Taking the spatial dimension as an example, the correlation coefficient can be used to measure the correlation degree between the two channels, which can be expressed as

$C_{s_{i}s_{j}}=\frac{\int(I(t,s_{i})-\left\langle I(t,s_{i}) \right\rangle)(I(t,s_{j})-\left\langle I(t,s_{j}) \right\rangle)dt}{\sqrt{\int(I(t,s_{i})-\left\langle I(t,s_{i}) \right\rangle)^{2}dt}\sqrt{\int(I(t,s_{j})-\left\langle I(t,s_{j}) \right\rangle)^{2}dt}}$ (16)

where $\left\langle I(t,s_{i}) \right\rangle$ is the average value on *i*-th channel. After setting a target value of the total correlation coefficient $C_{T}$ for the event data, the correlation limit function added to the loss function can be expressed as

$(\sum_{i} \sum_{j} C_{s_{i}s_{j}}-C_{T})^{2}$ (17)

Figure S6 shows the shake data generated by PIGN with and without the use of the correlation constraint function. It can be observed that the data generated using the correlation constraint function exhibits correlation between the spatial channels. Based on actual conditions, similar correlation constraint function can be constructed in the frequency domain and time domain.

## 1.6 DAS constraint function

Since the DAS signal is a differential phase signal within the ± $\pi$ range, this characteristic needs to be fully considered in PIGN. For visualization purposes in the figures presented in the manuscript and the convenience of network training, we converted the phase values to the range of 0 to 2π. The direct method is to limit the value of PIGN output. The following activation function are performed at the last layer of PIGN:

$2\pi Sigmoid(x)$ (18)

Although this method can make the output value of PIGN within 0 to 2π, it can not make the PIGN learn the characteristics of phase wrapping in DAS data. To overcome this problem, we combine the phase wrapping function with the target event feature function to make the network learn the characteristics of the phase entangled DAS signal. Taking the time-domain feature function as an example, the phase entangled temporal feature function $T_{wrapped}$ is obtained by performing phase wrapping operation on the temporal feature function $T$, which can be served as a type of DAS constraint function and expressed as

$D\left( T\left( t \right) \right)= T_{wrapped}=T(t)-2\pi\left[ \frac{T(t)+\pi}{2\pi} \right]$ (19)

where $\left[ \cdot\right]$ is the floor function.

# 2. Physics-informed generative network, PIGN

## 2.1 Full loss function

Considering the above constraint functions, the final full loss function for PIGN can be expressed as follows:

$W_{0}(\left\|\widetilde{T}\text{-}D(T_{Event})\right\|^{2}+\left\|\widetilde{S}\text{-}D(S_{Event})\right\|^{2})+\sum_{n} W_{\text{n}}R_{n}$ (20)

Where *W* stands for weight ($W_{0}$ is always set to 1), $\widetilde{T}$and $\widetilde{S}$are the feature curves of PIGN output data in the time domain and space domain or frequency domain, $T_{Event}$ and $S_{Event}$ are the target feature curves obtained from feature functions, *D()* is the DAS constraint function (specifically the phase wrapping function here), $R$ is real-world constraint function (The specific equations and number of functions depend on the target event).

For all types of data (shake, walk, and belt conveyor fault), the continuous signal constraint function was used as a type of real-world constraint function $R_{1}$ during PIGN training, with the corresponding weight set to 0.01. For the shake data, an additional spatial correlation constraint function which acted as another type of real-world constraint function $R_{2}$ was applied, also with a weight of 0.01.

For public dataset, since the DAS data represents phase value, phase wrapping function is used as the DAS constraint function when calculating the full loss.

For belt conveyor applications, since our focus is on the time–frequency behavior of the signals, the phase-wrapping DAS constraint function is not applicable to the feature functions in these experiments. Here, the DAS constraint function simply becomes a constant factor of 1, i.e., it preserves the original feature function.

Since the primary goal of PIGN is to learn the event feature functions, the loss term corresponding to the target feature functions is always assigned the maximum weight of 1. The loss terms associated with the real-world constraint functions serve only to further adjust the PIGN-generated data to better match real-world data and can be regarded as a form of penalty or regularization. Here, we selected a relatively small weight of 0.01 for these real-world constraint terms to ensure that the network remains primarily focused on learning the feature functions. The choice of 0.01 follows commonly used recommendations for initializing weights in neural network training and was adopted solely as an initial setting. Since the network functioned properly with this weight parameter, we did not further pursue optimization of this value.

## 2.2 The training mode of PIGN


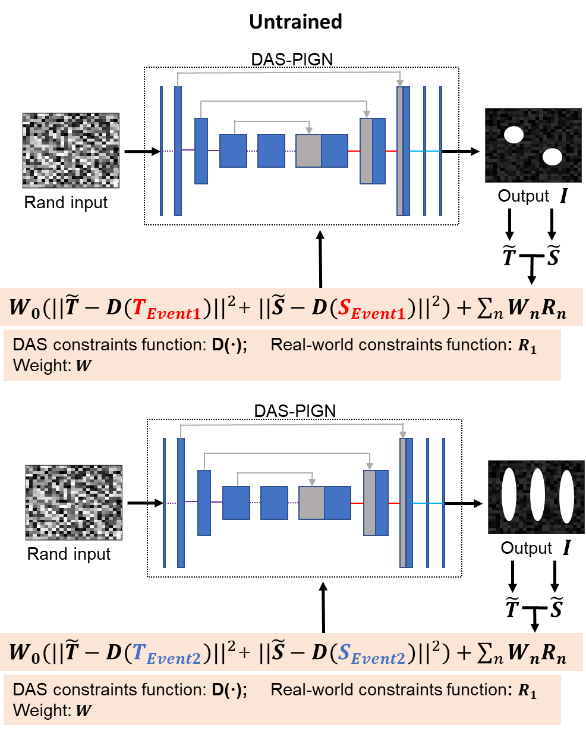


Figure S7 Schematic diagram of the training mode for PIGN.

PIGN adopts an untrained mode for training. Figure S7 shows the working process of PIGN in this training mode. The process begins with the construction of feature functions of the event, from which various feature curves of the event can be derived by specifying the parameters within the feature functions. In the untrained mode, the PIGN is trained using only a single set of feature curves as its target. Once the training is complete, the PIGN is capable of generating the corresponding DAS data. If we replace the feature curves with a new set (i.e., a different parameterization of the same feature functions), we can repeat the training process and obtain a different DAS data output. As illustrated in the Figure S7, in this mode, PIGN only takes a single random input image and generates an individual DAS data. Since PIGN only need to learn the feature curves of an individual event, the untrained mode is easy to reach convergence in the training process, and the generated data can have specific feature, making it suitable for small batch specific data generation.

## 2.3 PIGN structure

The detailed structure of PIGN is as follows:

PIGN(

(c1): Conv2d(1, 16, kernel_size=(3, 3), stride=(1, 1), padding=(1, 1), padding_mode=replicate)

(c1a): LeakyReLU(negative_slope=0.01)

(c2): Conv2d(16, 16, kernel_size=(3, 3), stride=(1, 1), padding=(1, 1), padding_mode=replicate)

(c2a): LeakyReLU(negative_slope=0.01)

(c2M): MaxPool2d(kernel_size=2, stride=2, padding=0, dilation=1, ceil_mode=False)

(c3): Conv2d(16, 32, kernel_size=(3, 3), stride=(1, 1), padding=(1, 1), padding_mode=replicate)

(c3a): LeakyReLU(negative_slope=0.01)

(c4): Conv2d(32, 32, kernel_size=(3, 3), stride=(1, 1), padding=(1, 1), padding_mode=replicate)

(c4a): LeakyReLU(negative_slope=0.01)

(c4M): MaxPool2d(kernel_size=2, stride=2, padding=0, dilation=1, ceil_mode=False)

(c5): Conv2d(32, 64, kernel_size=(3, 3), stride=(1, 1), padding=(1, 1), padding_mode=replicate)

(c5a): LeakyReLU(negative_slope=0.01)

(c6): Conv2d(64, 64, kernel_size=(3, 3), stride=(1, 1), padding=(1, 1), padding_mode=replicate)

(c6a): LeakyReLU(negative_slope=0.01)

(c6M): MaxPool2d(kernel_size=2, stride=2, padding=0, dilation=1, ceil_mode=False)

(c7): Conv2d(64, 128, kernel_size=(3, 3), stride=(1, 1), padding=(1, 1), padding_mode=replicate)

(c7a): LeakyReLU(negative_slope=0.01)

(c8): Conv2d(128, 128, kernel_size=(3, 3), stride=(1, 1), padding=(1, 1), padding_mode=replicate)

(c8a): LeakyReLU(negative_slope=0.01)

(c8M): MaxPool2d(kernel_size=2, stride=2, padding=0, dilation=1, ceil_mode=False)

(c71): Conv2d(128, 256, kernel_size=(3, 3), stride=(1, 1), padding=(1, 1), padding_mode=replicate)

(c71a): LeakyReLU(negative_slope=0.01)

(c81): Conv2d(256, 256, kernel_size=(3, 3), stride=(1, 1), padding=(1, 1), padding_mode=replicate)

(c81a): LeakyReLU(negative_slope=0.01)

(uc91): ConvTranspose2d(256, 128, kernel_size=(2, 2), stride=(2, 2), output_padding=(0, 1))

(uc91a): LeakyReLU(negative_slope=0.01)

(c101): Conv2d(256, 128, kernel_size=(3, 3), stride=(1, 1), padding=(1, 1), padding_mode=replicate)

(c101a): LeakyReLU(negative_slope=0.01)

(c111): Conv2d(128, 128, kernel_size=(3, 3), stride=(1, 1), padding=(1, 1), padding_mode=replicate)

(c111a): LeakyReLU(negative_slope=0.01)

(uc9): ConvTranspose2d(128, 64, kernel_size=(2, 2), stride=(2, 2))

(uc9a): LeakyReLU(negative_slope=0.01)

(c10): Conv2d(128, 64, kernel_size=(3, 3), stride=(1, 1), padding=(1, 1), padding_mode=replicate)

(c10a): LeakyReLU(negative_slope=0.01)

(c11): Conv2d(64, 64, kernel_size=(3, 3), stride=(1, 1), padding=(1, 1), padding_mode=replicate)

(c11a): LeakyReLU(negative_slope=0.01)

(uc12): ConvTranspose2d(64, 32, kernel_size=(2, 2), stride=(2, 2))

(uc12a): LeakyReLU(negative_slope=0.01)

(c13): Conv2d(64, 32, kernel_size=(3, 3), stride=(1, 1), padding=(1, 1), padding_mode=replicate)

(c13a): LeakyReLU(negative_slope=0.01)

(c14): Conv2d(32, 32, kernel_size=(3, 3), stride=(1, 1), padding=(1, 1), padding_mode=replicate)

(c14a): LeakyReLU(negative_slope=0.01)

(uc15): ConvTranspose2d(32, 16, kernel_size=(2, 2), stride=(2, 2), output_padding=(1, 0))

(uc15a): LeakyReLU(negative_slope=0.01)

(c15_5): Conv2d(32, 32, kernel_size=(3, 3), stride=(1, 1), padding=(1, 1), padding_mode=replicate)

(c15a): LeakyReLU(negative_slope=0.01)

(c16): Conv2d(32, 1, kernel_size=(3, 3), stride=(1, 1), padding=(1, 1), padding_mode=replicate)

(c16a): Sigmoid()

)

Because the network outputs are constrained to a fixed numerical range, a sigmoid activation was used in the final layer. It should be noted that for DAS data of walk and shake within 0 to 2π, the activation function of the network’s last layer should be multiplied by 2π to ensure the output is in the range of 0 to 2π.

The information of relevant hyperparameters is as follows:

**1. For public dataset:**

|  | **PIGN**  **(trained mode)** | **PIGN**  **(untrained mode)** |
| --- | --- | --- |
| Optimization algorithm | Adam | Adam |
| Learning rate | 1e-5 | 1e-5 |
| Maximum epochs | 40000 | 10000 |
| batchsize | 4 | 1 |
| Weight initialization | kaiming_uniform | kaiming_uniform |

**2. For belt conveyor fault data generation:**

|  | **PIGN**  **(trained mode)** | **PIGN**  **(untrained mode)** |
| --- | --- | --- |
| Optimization algorithm | Adam | Adam |
| Learning rate | 1e-5 | 1e-5 |
| Maximum epochs | 2500 | 15000 |
| batchsize | 4 | 1 |
| Weight initialization | kaiming_uniform | kaiming_uniform |

## 2.4 Generated shake and walk data by PIGN vs real-world data

Some real background data examples are shown below:

Figure S8 Real background data in public dataset.

Here we show the comparison between PIGN generated data and real data from public dataset.

Figure S9 Real-world shake data in public dataset (Left side) and generated shake data by PIGN (Right side).

Figure S10 Real-world walk data in public dataset (Left side) and generated walk data by PIGN (Right side).

As the training of the PIGN model relies on physical model, the generated data is designed to capture the general characteristics of real-world event data rather than replicate its exact visual appearance. Currently available image similarity metrics mainly assess structural similarities at the pixel or image level, which are not suitable for our scenario. This is because the generated data and real-world data do not resemble each other in terms of visual structure. Instead, their similarity lies in the presence of characteristic event-related signal patterns, which may manifest differently across individual data instances. At this stage, such similarity can only be qualitatively assessed based on human observation of the shared signal patterns. For example, both the real-world and generated data for shake events exhibit many spike-like signals in the time domain, whereas walk event signals show quasi-sinusoidal patterns in both real-world and generated data. In addition, the similarity between PIGN-generated data and RWD has been demonstrated in the classification results.

Phase wrapping can be observed in the time-domain feature curves of the first, second, and third PIGN-generated walk data on the right side of Figure S10, indicating that PIGN can generate data consistent with real DAS signals.

## 2.5 Generated belt conveyor fault data by PIGN vs real-world data

Figure S11 Real-world background data of belt conveyor simulation test site.

Figure S12 Real-world background data of belt conveyor in coal mine field site.

During normal operation, belt conveyors generate intense low-frequency mechanical vibrations, which result in strong signal energy concentrated within the sub-1 kHz frequency range in the DAS data as shown in Figure S11 and S12. However, during normal operation, some rollers can exhibit transient time-domain impact-like features that resemble those of faulty rollers. Additionally, aged rollers or rollers under suboptimal working conditions (though not yet classified as faulty) may occasionally present sparse-frequency or broadband-frequency characteristics in the spectral domain, similar to actual fault signals. These phenomena are specifically illustrated in Figure S11 and Figure S12 where the dashed circles highlight such cases. The red dotted line is the time domain impact feature, the blue dotted line is the sparse frequency feature, and the green dotted line is the broadband frequency feature. Therefore, it is difficult to eliminate background noise and retain fault signals. In addition, the background signals vary between different sites and at different locations within the same site, which greatly increases the difficulty of algorithm generalization.

Figure S13 Real-world fault data of belt conveyor simulation test site (left side) and coal mine field site (right side).

From Figure S13, it can be seen that the frequency domain of fault signals is mainly divided into two classes: sparse frequency feature and broadband frequency feature. For example, the first picture on the left side shows a broadband-frequency fault signal, characterized by significant energy distributed across a wide frequency range from 1 kHz to 15 kHz. When combined with the impulsive features in the time domain, such signals typically appear as vertical stripe patterns in the time–frequency spectrogram (as indicated by the red dashed circle). In the corresponding frequency-domain feature curve, relatively high values can be observed across most of the frequency range (values within the red dashed circle are generally above 0.05). In contrast, the first picture on the right side depicts a sparse-frequency fault signal, which exhibits strong energy concentrated at one or several specific frequency components (distinct from the low-frequency noise below 1 kHz caused by normal belt conveyor operation). In the time–frequency spectrogram, this signal type shows intermittent, high-energy patterns localized at certain frequency bands (as indicated by the blue dashed circle), while most other frequency regions remain at low energy, appearing nearly white in the grayscale spectrogram. Correspondingly, in the frequency-domain feature curve, prominent peaks appear above 1 kHz, which is beyond the low-frequency interference region commonly associated with conveyor operation (as indicated by the blue dashed circle). It is worth noting that both fault types share a common feature in the time domain: they exhibit relatively strong impulsive signatures in the time-domain feature curve.

Figure S14 Results of fault data generated by PIGN and the sum of real-world background data and generated fault data.

In Figure S14, the data are normalized for comparison. To better compare the PIGN-generated data with real data, Figure S15 shows both generated and RWD for the two fault types.

Figure S15 Belt conveyor fault data generated by PIGN (left side) and actual belt conveyor fault data (right side)

# 3. Noise-removal net

## 3.1 Principle and Structure of noise-removal net

In the training process of the noise-removal network, the input consists of the sum of real-world background data (B) and generated target event data (S), i.e., (B + S), while the corresponding label is the real-world background data (B). As illustrated in Figure 4e, the noise-removal network learns to extract the real background component (B) from the input (B + S).

After training, when real-world data (B_r + S_r) is input into the trained noise-removal network, the network extracts the corresponding background component (B_r). By subtracting this estimated background from the original input, we obtain the background-removed target event signal (S_r = (B_r + S_r) − B_r), as shown in Figure 4f. Additionally, we applied a ReLU operation to the resulting signal after subtraction. This is because the background data extracted by the network occasionally exceeds the original input at certain data points, resulting in negative values of the background-removed target event signal. The ReLU operation ensures these negative values are set to zero while retaining the meaningful positive components.

DenoisedNet(

(c1): Conv2d(1, 16, kernel_size=(3, 3), stride=(1, 1), padding=(1, 1), padding_mode=replicate)

(c1a): LeakyReLU(negative_slope=0.01)

(c2): Conv2d(16, 16, kernel_size=(3, 3), stride=(1, 1), padding=(1, 1), padding_mode=replicate)

(c2a): LeakyReLU(negative_slope=0.01)

(c2M): MaxPool2d(kernel_size=2, stride=2, padding=0, dilation=1, ceil_mode=False)

(c3): Conv2d(16, 32, kernel_size=(3, 3), stride=(1, 1), padding=(1, 1), padding_mode=replicate)

(c3a): LeakyReLU(negative_slope=0.01)

(c4): Conv2d(32, 32, kernel_size=(3, 3), stride=(1, 1), padding=(1, 1), padding_mode=replicate)

(c4a): LeakyReLU(negative_slope=0.01)

(c4M): MaxPool2d(kernel_size=2, stride=2, padding=0, dilation=1, ceil_mode=False)

(c5): Conv2d(32, 64, kernel_size=(3, 3), stride=(1, 1), padding=(1, 1), padding_mode=replicate)

(c5a): LeakyReLU(negative_slope=0.01)

(c6): Conv2d(64, 64, kernel_size=(3, 3), stride=(1, 1), padding=(1, 1), padding_mode=replicate)

(c6a): LeakyReLU(negative_slope=0.01)

(c6M): MaxPool2d(kernel_size=2, stride=2, padding=0, dilation=1, ceil_mode=False)

(c7): Conv2d(64, 128, kernel_size=(3, 3), stride=(1, 1), padding=(1, 1), padding_mode=replicate)

(c7a): LeakyReLU(negative_slope=0.01)

(c8): Conv2d(128, 128, kernel_size=(3, 3), stride=(1, 1), padding=(1, 1), padding_mode=replicate)

(c8a): LeakyReLU(negative_slope=0.01)

(c8M): MaxPool2d(kernel_size=2, stride=2, padding=0, dilation=1, ceil_mode=False)

(c71): Conv2d(128, 256, kernel_size=(3, 3), stride=(1, 1), padding=(1, 1), padding_mode=replicate)

(c71a): LeakyReLU(negative_slope=0.01)

(c81): Conv2d(256, 256, kernel_size=(3, 3), stride=(1, 1), padding=(1, 1), padding_mode=replicate)

(c81a): LeakyReLU(negative_slope=0.01)

(uc91): ConvTranspose2d(256, 128, kernel_size=(2, 2), stride=(2, 2), output_padding=(0, 1))

(uc91a): LeakyReLU(negative_slope=0.01)

(c101): Conv2d(256, 128, kernel_size=(3, 3), stride=(1, 1), padding=(1, 1), padding_mode=replicate)

(c101a): LeakyReLU(negative_slope=0.01)

(c111): Conv2d(128, 128, kernel_size=(3, 3), stride=(1, 1), padding=(1, 1), padding_mode=replicate)

(c111a): LeakyReLU(negative_slope=0.01)

(uc9): ConvTranspose2d(128, 64, kernel_size=(2, 2), stride=(2, 2))

(uc9a): LeakyReLU(negative_slope=0.01)

(c10): Conv2d(128, 64, kernel_size=(3, 3), stride=(1, 1), padding=(1, 1), padding_mode=replicate)

(c10a): LeakyReLU(negative_slope=0.01)

(c11): Conv2d(64, 64, kernel_size=(3, 3), stride=(1, 1), padding=(1, 1), padding_mode=replicate)

(c11a): LeakyReLU(negative_slope=0.01)

(uc12): ConvTranspose2d(64, 32, kernel_size=(2, 2), stride=(2, 2))

(uc12a): LeakyReLU(negative_slope=0.01)

(c13): Conv2d(64, 32, kernel_size=(3, 3), stride=(1, 1), padding=(1, 1), padding_mode=replicate)

(c13a): LeakyReLU(negative_slope=0.01)

(c14): Conv2d(32, 32, kernel_size=(3, 3), stride=(1, 1), padding=(1, 1), padding_mode=replicate)

(c14a): LeakyReLU(negative_slope=0.01)

(uc15): ConvTranspose2d(32, 16, kernel_size=(2, 2), stride=(2, 2), output_padding=(1, 0))

(uc15a): LeakyReLU(negative_slope=0.01)

(c15_5): Conv2d(32, 32, kernel_size=(3, 3), stride=(1, 1), padding=(1, 1), padding_mode=replicate)

(c15a): LeakyReLU(negative_slope=0.01)

(c16): Conv2d(32, 1, kernel_size=(3, 3), stride=(1, 1), padding=(1, 1), padding_mode=replicate)

(c16a): Sigmoid()

)

The hyperparameter setting:

|  | **Noise-removal net** |
| --- | --- |
| Optimization algorithm | Adam |
| Learning rate | 1e-5 |
| Maximum epochs | 150 |
| batchsize | 8 |
| Weight initialization | kaiming_uniform |

## 3.2 Denoised results

Figure S16 The results before and after denoised of real-world background data.

Figure S17 The results before and after denoised of real-world fault data.

The noise-removal net is trained by generated fault data and the real-world background data of belt conveyor simulation test site, so the background removal effect is better in the simulation test site. Since the background noise generation mechanism of belt conveyor simulation test site and coal mine field site is similar, the background noise of both sites share similar features. Therefore, when the noise-removal net is directly applied to the coal mine field, the background noise of the field data can also be removed, as shown in Figure S16 and S17. However, denoised data of coal mine field still retains some characteristic signals due to some specific characteristics in the signals between different sites. In general, since the noise-removal net has learned the common characteristics of background noise in the application, it can still achieve effective background noise removal when directly applied to different sites.

# 4. Classification networks

## 4.1 Structures of classification networks

**The structure of CNN for shake and walk recognition is as follows:**

CNN(

(conv1): Sequential(

(0): Conv2d(1, 5, kernel_size=(20, 3), stride=(50, 1), padding=(1, 1))

(1): ReLU()

(2): MaxPool2d(kernel_size=2, stride=2, padding=1, dilation=1, ceil_mode=False)

)

(conv2): Sequential(

(0): Conv2d(5, 10, kernel_size=(2, 2), stride=(4, 1), padding=(1, 1))

(1): ReLU()

(2): MaxPool2d(kernel_size=2, stride=2, padding=0, dilation=1, ceil_mode=False)

)

(out): Linear(in_features=40, out_features=3, bias=True)

)

**The structure of CNN for belt conveyor fault monitoring is as follows:**

CNN(

(conv1): Sequential(

(0): Conv2d(1, 8, kernel_size=(6, 3), stride=(2, 1), padding=(1, 1))

(1): ReLU()

(2): MaxPool2d(kernel_size=2, stride=2, padding=1, dilation=1, ceil_mode=False)

)

(conv2): Sequential(

(0): Conv2d(8, 8, kernel_size=(3, 3), stride=(2, 1), padding=(1, 1))

(1): ReLU()

(2): MaxPool2d(kernel_size=2, stride=2, padding=0, dilation=1, ceil_mode=False)

)

(out): Linear(in_features=74752, out_features=3, bias=True)

)

**The structure of ResNet34 for shake and walk recognition is as follows:**

ResNet(

(conv1): Sequential(

(0): Conv2d(1, 64, kernel_size=(7, 7), stride=(2, 2), padding=(3, 3))

(1): BatchNorm2d(64, eps=1e-05, momentum=0.1, affine=True, track_running_stats=True)

(2): ReLU()

)

(maxpool): MaxPool2d(kernel_size=3, stride=2, padding=1, dilation=1, ceil_mode=False)

(layer0): Sequential(

(0): ResidualBlock(

(conv1): Sequential(

(0): Conv2d(64, 64, kernel_size=(3, 3), stride=(1, 1), padding=(1, 1))

(1): BatchNorm2d(64, eps=1e-05, momentum=0.1, affine=True, track_running_stats=True)

(2): ReLU()

)

(conv2): Sequential(

(0): Conv2d(64, 64, kernel_size=(3, 3), stride=(1, 1), padding=(1, 1))

(1): BatchNorm2d(64, eps=1e-05, momentum=0.1, affine=True, track_running_stats=True)

)

(relu): ReLU()

)

(1): ResidualBlock(

(conv1): Sequential(

(0): Conv2d(64, 64, kernel_size=(3, 3), stride=(1, 1), padding=(1, 1))

(1): BatchNorm2d(64, eps=1e-05, momentum=0.1, affine=True, track_running_stats=True)

(2): ReLU()

)

(conv2): Sequential(

(0): Conv2d(64, 64, kernel_size=(3, 3), stride=(1, 1), padding=(1, 1))

(1): BatchNorm2d(64, eps=1e-05, momentum=0.1, affine=True, track_running_stats=True)

)

(relu): ReLU()

)

(2): ResidualBlock(

(conv1): Sequential(

(0): Conv2d(64, 64, kernel_size=(3, 3), stride=(1, 1), padding=(1, 1))

(1): BatchNorm2d(64, eps=1e-05, momentum=0.1, affine=True, track_running_stats=True)

(2): ReLU()

)

(conv2): Sequential(

(0): Conv2d(64, 64, kernel_size=(3, 3), stride=(1, 1), padding=(1, 1))

(1): BatchNorm2d(64, eps=1e-05, momentum=0.1, affine=True, track_running_stats=True)

)

(relu): ReLU()

)

)

(layer1): Sequential(

(0): ResidualBlock(

(conv1): Sequential(

(0): Conv2d(64, 128, kernel_size=(3, 3), stride=(2, 2), padding=(1, 1))

(1): BatchNorm2d(128, eps=1e-05, momentum=0.1, affine=True, track_running_stats=True)

(2): ReLU()

)

(conv2): Sequential(

(0): Conv2d(128, 128, kernel_size=(3, 3), stride=(1, 1), padding=(1, 1))

(1): BatchNorm2d(128, eps=1e-05, momentum=0.1, affine=True, track_running_stats=True)

)

(downsample): Sequential(

(0): Conv2d(64, 128, kernel_size=(1, 1), stride=(2, 2))

(1): BatchNorm2d(128, eps=1e-05, momentum=0.1, affine=True, track_running_stats=True)

)

(relu): ReLU()

)

(1): ResidualBlock(

(conv1): Sequential(

(0): Conv2d(128, 128, kernel_size=(3, 3), stride=(1, 1), padding=(1, 1))

(1): BatchNorm2d(128, eps=1e-05, momentum=0.1, affine=True, track_running_stats=True)

(2): ReLU()

)

(conv2): Sequential(

(0): Conv2d(128, 128, kernel_size=(3, 3), stride=(1, 1), padding=(1, 1))

(1): BatchNorm2d(128, eps=1e-05, momentum=0.1, affine=True, track_running_stats=True)

)

(relu): ReLU()

)

(2): ResidualBlock(

(conv1): Sequential(

(0): Conv2d(128, 128, kernel_size=(3, 3), stride=(1, 1), padding=(1, 1))

(1): BatchNorm2d(128, eps=1e-05, momentum=0.1, affine=True, track_running_stats=True)

(2): ReLU()

)

(conv2): Sequential(

(0): Conv2d(128, 128, kernel_size=(3, 3), stride=(1, 1), padding=(1, 1))

(1): BatchNorm2d(128, eps=1e-05, momentum=0.1, affine=True, track_running_stats=True)

)

(relu): ReLU()

)

(3): ResidualBlock(

(conv1): Sequential(

(0): Conv2d(128, 128, kernel_size=(3, 3), stride=(1, 1), padding=(1, 1))

(1): BatchNorm2d(128, eps=1e-05, momentum=0.1, affine=True, track_running_stats=True)

(2): ReLU()

)

(conv2): Sequential(

(0): Conv2d(128, 128, kernel_size=(3, 3), stride=(1, 1), padding=(1, 1))

(1): BatchNorm2d(128, eps=1e-05, momentum=0.1, affine=True, track_running_stats=True)

)

(relu): ReLU()

)

)

(layer2): Sequential(

(0): ResidualBlock(

(conv1): Sequential(

(0): Conv2d(128, 256, kernel_size=(3, 3), stride=(2, 2), padding=(1, 1))

(1): BatchNorm2d(256, eps=1e-05, momentum=0.1, affine=True, track_running_stats=True)

(2): ReLU()

)

(conv2): Sequential(

(0): Conv2d(256, 256, kernel_size=(3, 3), stride=(1, 1), padding=(1, 1))

(1): BatchNorm2d(256, eps=1e-05, momentum=0.1, affine=True, track_running_stats=True)

)

(downsample): Sequential(

(0): Conv2d(128, 256, kernel_size=(1, 1), stride=(2, 2))

(1): BatchNorm2d(256, eps=1e-05, momentum=0.1, affine=True, track_running_stats=True)

)

(relu): ReLU()

)

(1): ResidualBlock(

(conv1): Sequential(

(0): Conv2d(256, 256, kernel_size=(3, 3), stride=(1, 1), padding=(1, 1))

(1): BatchNorm2d(256, eps=1e-05, momentum=0.1, affine=True, track_running_stats=True)

(2): ReLU()

)

(conv2): Sequential(

(0): Conv2d(256, 256, kernel_size=(3, 3), stride=(1, 1), padding=(1, 1))

(1): BatchNorm2d(256, eps=1e-05, momentum=0.1, affine=True, track_running_stats=True)

)

(relu): ReLU()

)

(2): ResidualBlock(

(conv1): Sequential(

(0): Conv2d(256, 256, kernel_size=(3, 3), stride=(1, 1), padding=(1, 1))

(1): BatchNorm2d(256, eps=1e-05, momentum=0.1, affine=True, track_running_stats=True)

(2): ReLU()

)

(conv2): Sequential(

(0): Conv2d(256, 256, kernel_size=(3, 3), stride=(1, 1), padding=(1, 1))

(1): BatchNorm2d(256, eps=1e-05, momentum=0.1, affine=True, track_running_stats=True)

)

(relu): ReLU()

)

(3): ResidualBlock(

(conv1): Sequential(

(0): Conv2d(256, 256, kernel_size=(3, 3), stride=(1, 1), padding=(1, 1))

(1): BatchNorm2d(256, eps=1e-05, momentum=0.1, affine=True, track_running_stats=True)

(2): ReLU()

)

(conv2): Sequential(

(0): Conv2d(256, 256, kernel_size=(3, 3), stride=(1, 1), padding=(1, 1))

(1): BatchNorm2d(256, eps=1e-05, momentum=0.1, affine=True, track_running_stats=True)

)

(relu): ReLU()

)

(4): ResidualBlock(

(conv1): Sequential(

(0): Conv2d(256, 256, kernel_size=(3, 3), stride=(1, 1), padding=(1, 1))

(1): BatchNorm2d(256, eps=1e-05, momentum=0.1, affine=True, track_running_stats=True)

(2): ReLU()

)

(conv2): Sequential(

(0): Conv2d(256, 256, kernel_size=(3, 3), stride=(1, 1), padding=(1, 1))

(1): BatchNorm2d(256, eps=1e-05, momentum=0.1, affine=True, track_running_stats=True)

)

(relu): ReLU()

)

(5): ResidualBlock(

(conv1): Sequential(

(0): Conv2d(256, 256, kernel_size=(3, 3), stride=(1, 1), padding=(1, 1))

(1): BatchNorm2d(256, eps=1e-05, momentum=0.1, affine=True, track_running_stats=True)

(2): ReLU()

)

(conv2): Sequential(

(0): Conv2d(256, 256, kernel_size=(3, 3), stride=(1, 1), padding=(1, 1))

(1): BatchNorm2d(256, eps=1e-05, momentum=0.1, affine=True, track_running_stats=True)

)

(relu): ReLU()

)

)

(layer3): Sequential(

(0): ResidualBlock(

(conv1): Sequential(

(0): Conv2d(256, 512, kernel_size=(3, 3), stride=(2, 2), padding=(1, 1))

(1): BatchNorm2d(512, eps=1e-05, momentum=0.1, affine=True, track_running_stats=True)

(2): ReLU()

)

(conv2): Sequential(

(0): Conv2d(512, 512, kernel_size=(3, 3), stride=(1, 1), padding=(1, 1))

(1): BatchNorm2d(512, eps=1e-05, momentum=0.1, affine=True, track_running_stats=True)

)

(downsample): Sequential(

(0): Conv2d(256, 512, kernel_size=(1, 1), stride=(2, 2))

(1): BatchNorm2d(512, eps=1e-05, momentum=0.1, affine=True, track_running_stats=True)

)

(relu): ReLU()

)

(1): ResidualBlock(

(conv1): Sequential(

(0): Conv2d(512, 512, kernel_size=(3, 3), stride=(1, 1), padding=(1, 1))

(1): BatchNorm2d(512, eps=1e-05, momentum=0.1, affine=True, track_running_stats=True)

(2): ReLU()

)

(conv2): Sequential(

(0): Conv2d(512, 512, kernel_size=(3, 3), stride=(1, 1), padding=(1, 1))

(1): BatchNorm2d(512, eps=1e-05, momentum=0.1, affine=True, track_running_stats=True)

)

(relu): ReLU()

)

(2): ResidualBlock(

(conv1): Sequential(

(0): Conv2d(512, 512, kernel_size=(3, 3), stride=(1, 1), padding=(1, 1))

(1): BatchNorm2d(512, eps=1e-05, momentum=0.1, affine=True, track_running_stats=True)

(2): ReLU()

)

(conv2): Sequential(

(0): Conv2d(512, 512, kernel_size=(3, 3), stride=(1, 1), padding=(1, 1))

(1): BatchNorm2d(512, eps=1e-05, momentum=0.1, affine=True, track_running_stats=True)

)

(relu): ReLU()

)

)

(fc): Linear(in_features=16384, out_features=3, bias=True)

)

**The structure of ResNet34 for belt conveyor fault monitoring is as follows:**

ResNet(

(conv1): Sequential(

(0): Conv2d(1, 64, kernel_size=(7, 7), stride=(2, 2), padding=(3, 3))

(1): BatchNorm2d(64, eps=1e-05, momentum=0.1, affine=True, track_running_stats=True)

(2): ReLU()

)

(maxpool): MaxPool2d(kernel_size=3, stride=2, padding=1, dilation=1, ceil_mode=False)

(layer0): Sequential(

(0): ResidualBlock(

(conv1): Sequential(

(0): Conv2d(64, 64, kernel_size=(3, 3), stride=(1, 1), padding=(1, 1))

(1): BatchNorm2d(64, eps=1e-05, momentum=0.1, affine=True, track_running_stats=True)

(2): ReLU()

)

(conv2): Sequential(

(0): Conv2d(64, 64, kernel_size=(3, 3), stride=(1, 1), padding=(1, 1))

(1): BatchNorm2d(64, eps=1e-05, momentum=0.1, affine=True, track_running_stats=True)

)

(relu): ReLU()

)

(1): ResidualBlock(

(conv1): Sequential(

(0): Conv2d(64, 64, kernel_size=(3, 3), stride=(1, 1), padding=(1, 1))

(1): BatchNorm2d(64, eps=1e-05, momentum=0.1, affine=True, track_running_stats=True)

(2): ReLU()

)

(conv2): Sequential(

(0): Conv2d(64, 64, kernel_size=(3, 3), stride=(1, 1), padding=(1, 1))

(1): BatchNorm2d(64, eps=1e-05, momentum=0.1, affine=True, track_running_stats=True)

)

(relu): ReLU()

)

(2): ResidualBlock(

(conv1): Sequential(

(0): Conv2d(64, 64, kernel_size=(3, 3), stride=(1, 1), padding=(1, 1))

(1): BatchNorm2d(64, eps=1e-05, momentum=0.1, affine=True, track_running_stats=True)

(2): ReLU()

)

(conv2): Sequential(

(0): Conv2d(64, 64, kernel_size=(3, 3), stride=(1, 1), padding=(1, 1))

(1): BatchNorm2d(64, eps=1e-05, momentum=0.1, affine=True, track_running_stats=True)

)

(relu): ReLU()

)

)

(layer1): Sequential(

(0): ResidualBlock(

(conv1): Sequential(

(0): Conv2d(64, 128, kernel_size=(3, 3), stride=(2, 2), padding=(1, 1))

(1): BatchNorm2d(128, eps=1e-05, momentum=0.1, affine=True, track_running_stats=True)

(2): ReLU()

)

(conv2): Sequential(

(0): Conv2d(128, 128, kernel_size=(3, 3), stride=(1, 1), padding=(1, 1))

(1): BatchNorm2d(128, eps=1e-05, momentum=0.1, affine=True, track_running_stats=True)

)

(downsample): Sequential(

(0): Conv2d(64, 128, kernel_size=(1, 1), stride=(2, 2))

(1): BatchNorm2d(128, eps=1e-05, momentum=0.1, affine=True, track_running_stats=True)

)

(relu): ReLU()

)

(1): ResidualBlock(

(conv1): Sequential(

(0): Conv2d(128, 128, kernel_size=(3, 3), stride=(1, 1), padding=(1, 1))

(1): BatchNorm2d(128, eps=1e-05, momentum=0.1, affine=True, track_running_stats=True)

(2): ReLU()

)

(conv2): Sequential(

(0): Conv2d(128, 128, kernel_size=(3, 3), stride=(1, 1), padding=(1, 1))

(1): BatchNorm2d(128, eps=1e-05, momentum=0.1, affine=True, track_running_stats=True)

)

(relu): ReLU()

)

(2): ResidualBlock(

(conv1): Sequential(

(0): Conv2d(128, 128, kernel_size=(3, 3), stride=(1, 1), padding=(1, 1))

(1): BatchNorm2d(128, eps=1e-05, momentum=0.1, affine=True, track_running_stats=True)

(2): ReLU()

)

(conv2): Sequential(

(0): Conv2d(128, 128, kernel_size=(3, 3), stride=(1, 1), padding=(1, 1))

(1): BatchNorm2d(128, eps=1e-05, momentum=0.1, affine=True, track_running_stats=True)

)

(relu): ReLU()

)

(3): ResidualBlock(

(conv1): Sequential(

(0): Conv2d(128, 128, kernel_size=(3, 3), stride=(1, 1), padding=(1, 1))

(1): BatchNorm2d(128, eps=1e-05, momentum=0.1, affine=True, track_running_stats=True)

(2): ReLU()

)

(conv2): Sequential(

(0): Conv2d(128, 128, kernel_size=(3, 3), stride=(1, 1), padding=(1, 1))

(1): BatchNorm2d(128, eps=1e-05, momentum=0.1, affine=True, track_running_stats=True)

)

(relu): ReLU()

)

)

(layer2): Sequential(

(0): ResidualBlock(

(conv1): Sequential(

(0): Conv2d(128, 256, kernel_size=(3, 3), stride=(2, 2), padding=(1, 1))

(1): BatchNorm2d(256, eps=1e-05, momentum=0.1, affine=True, track_running_stats=True)

(2): ReLU()

)

(conv2): Sequential(

(0): Conv2d(256, 256, kernel_size=(3, 3), stride=(1, 1), padding=(1, 1))

(1): BatchNorm2d(256, eps=1e-05, momentum=0.1, affine=True, track_running_stats=True)

)

(downsample): Sequential(

(0): Conv2d(128, 256, kernel_size=(1, 1), stride=(2, 2))

(1): BatchNorm2d(256, eps=1e-05, momentum=0.1, affine=True, track_running_stats=True)

)

(relu): ReLU()

)

(1): ResidualBlock(

(conv1): Sequential(

(0): Conv2d(256, 256, kernel_size=(3, 3), stride=(1, 1), padding=(1, 1))

(1): BatchNorm2d(256, eps=1e-05, momentum=0.1, affine=True, track_running_stats=True)

(2): ReLU()

)

(conv2): Sequential(

(0): Conv2d(256, 256, kernel_size=(3, 3), stride=(1, 1), padding=(1, 1))

(1): BatchNorm2d(256, eps=1e-05, momentum=0.1, affine=True, track_running_stats=True)

)

(relu): ReLU()

)

(2): ResidualBlock(

(conv1): Sequential(

(0): Conv2d(256, 256, kernel_size=(3, 3), stride=(1, 1), padding=(1, 1))

(1): BatchNorm2d(256, eps=1e-05, momentum=0.1, affine=True, track_running_stats=True)

(2): ReLU()

)

(conv2): Sequential(

(0): Conv2d(256, 256, kernel_size=(3, 3), stride=(1, 1), padding=(1, 1))

(1): BatchNorm2d(256, eps=1e-05, momentum=0.1, affine=True, track_running_stats=True)

)

(relu): ReLU()

)

(3): ResidualBlock(

(conv1): Sequential(

(0): Conv2d(256, 256, kernel_size=(3, 3), stride=(1, 1), padding=(1, 1))

(1): BatchNorm2d(256, eps=1e-05, momentum=0.1, affine=True, track_running_stats=True)

(2): ReLU()

)

(conv2): Sequential(

(0): Conv2d(256, 256, kernel_size=(3, 3), stride=(1, 1), padding=(1, 1))

(1): BatchNorm2d(256, eps=1e-05, momentum=0.1, affine=True, track_running_stats=True)

)

(relu): ReLU()

)

(4): ResidualBlock(

(conv1): Sequential(

(0): Conv2d(256, 256, kernel_size=(3, 3), stride=(1, 1), padding=(1, 1))

(1): BatchNorm2d(256, eps=1e-05, momentum=0.1, affine=True, track_running_stats=True)

(2): ReLU()

)

(conv2): Sequential(

(0): Conv2d(256, 256, kernel_size=(3, 3), stride=(1, 1), padding=(1, 1))

(1): BatchNorm2d(256, eps=1e-05, momentum=0.1, affine=True, track_running_stats=True)

)

(relu): ReLU()

)

(5): ResidualBlock(

(conv1): Sequential(

(0): Conv2d(256, 256, kernel_size=(3, 3), stride=(1, 1), padding=(1, 1))

(1): BatchNorm2d(256, eps=1e-05, momentum=0.1, affine=True, track_running_stats=True)

(2): ReLU()

)

(conv2): Sequential(

(0): Conv2d(256, 256, kernel_size=(3, 3), stride=(1, 1), padding=(1, 1))

(1): BatchNorm2d(256, eps=1e-05, momentum=0.1, affine=True, track_running_stats=True)

)

(relu): ReLU()

)

)

(layer3): Sequential(

(0): ResidualBlock(

(conv1): Sequential(

(0): Conv2d(256, 512, kernel_size=(3, 3), stride=(2, 2), padding=(1, 1))

(1): BatchNorm2d(512, eps=1e-05, momentum=0.1, affine=True, track_running_stats=True)

(2): ReLU()

)

(conv2): Sequential(

(0): Conv2d(512, 512, kernel_size=(3, 3), stride=(1, 1), padding=(1, 1))

(1): BatchNorm2d(512, eps=1e-05, momentum=0.1, affine=True, track_running_stats=True)

)

(downsample): Sequential(

(0): Conv2d(256, 512, kernel_size=(1, 1), stride=(2, 2))

(1): BatchNorm2d(512, eps=1e-05, momentum=0.1, affine=True, track_running_stats=True)

)

(relu): ReLU()

)

(1): ResidualBlock(

(conv1): Sequential(

(0): Conv2d(512, 512, kernel_size=(3, 3), stride=(1, 1), padding=(1, 1))

(1): BatchNorm2d(512, eps=1e-05, momentum=0.1, affine=True, track_running_stats=True)

(2): ReLU()

)

(conv2): Sequential(

(0): Conv2d(512, 512, kernel_size=(3, 3), stride=(1, 1), padding=(1, 1))

(1): BatchNorm2d(512, eps=1e-05, momentum=0.1, affine=True, track_running_stats=True)

)

(relu): ReLU()

)

(2): ResidualBlock(

(conv1): Sequential(

(0): Conv2d(512, 512, kernel_size=(3, 3), stride=(1, 1), padding=(1, 1))

(1): BatchNorm2d(512, eps=1e-05, momentum=0.1, affine=True, track_running_stats=True)

(2): ReLU()

)

(conv2): Sequential(

(0): Conv2d(512, 512, kernel_size=(3, 3), stride=(1, 1), padding=(1, 1))

(1): BatchNorm2d(512, eps=1e-05, momentum=0.1, affine=True, track_running_stats=True)

)

(relu): ReLU()

)

)

(avgpool): AvgPool2d(kernel_size=7, stride=1, padding=0)

(fc): Linear(in_features=179712, out_features=4, bias=True)

)

**The structure of CNN-BiLSTM for shake and walk recognition is as follows:**

CNN-BiLSTM(

(first_conv): Conv2d(1, 16, kernel_size=(125, 3), stride=(1, 1), padding=(124, 2), dilation=(2, 2))

(first_maxpool): MaxPool2d(kernel_size=(2, 1), stride=(2, 1), padding=0, dilation=1, ceil_mode=False)

(second_conv): Conv2d(16, 32, kernel_size=(60, 3), stride=(1, 1), padding=(59, 2), dilation=(2, 2))

(second_maxpool): MaxPool2d(kernel_size=(2, 1), stride=(2, 1), padding=0, dilation=1, ceil_mode=False)

(third_conv): Conv2d(32, 32, kernel_size=(30, 3), stride=(1, 1), padding=(29, 2), dilation=(2, 2))

(third_maxpool): MaxPool2d(kernel_size=(2, 1), stride=(2, 1), padding=0, dilation=1, ceil_mode=False)

(lstm_layer): LSTM(1500, 128, batch_first=True, bidirectional=True)

(first_dense): Linear(in_features=256, out_features=3, bias=True)

(first_ReLu): ReLU()

(second_ReLu): ReLU()

(third_ReLu): ReLU()

(softmax_layer): Softmax(dim=1)

)

**The structure of CNNBiLSTM for belt conveyor fault monitoring is as follows:**

CNNBiLSTM(

(first_conv): Conv2d(1, 16, kernel_size=(7, 3), stride=(1, 1), padding=(6, 2), dilation=(2, 2))

(first_maxpool): MaxPool2d(kernel_size=(2, 2), stride=(2, 2), padding=0, dilation=1, ceil_mode=False)

(second_conv): Conv2d(16, 32, kernel_size=(7, 3), stride=(1, 1), padding=(6, 2), dilation=(2, 2))

(second_maxpool): MaxPool2d(kernel_size=(2, 2), stride=(2, 2), padding=0, dilation=1, ceil_mode=False)

(third_conv): Conv2d(32, 32, kernel_size=(7, 3), stride=(1, 1), padding=(6, 2), dilation=(2, 2))

(third_maxpool): MaxPool2d(kernel_size=(2, 2), stride=(2, 2), padding=0, dilation=1, ceil_mode=False)

(lstm_layer): LSTM(9344, 512, batch_first=True, bidirectional=True)

(first_dense): Linear(in_features=1024, out_features=3, bias=True)

(first_ReLu): ReLU()

(second_ReLu): ReLU()

(third_ReLu): ReLU()

(softmax_layer): Softmax(dim=1)

)

**The hyperparameter settings for shake and walk data are:**

|  | **CNN** | **ResNet34** | **CNN-BiLSTM** |
| --- | --- | --- | --- |
| Optimization algorithm | Adam | Adam | Adam |
| Learning rate | 1e-3 | 1e-3 | 1e-3 |
| Maximum epochs | 500 | 500 | 500 |
| batchsize | 8 | 8 | 8 |
| Weight initialization | kaiming_uniform | kaiming_uniform | kaiming_uniform |

**The hyperparameter settings for belt conveyor fault monitoring data are:**

|  | **CNN** | **ResNet34** | **CNN-BiLSTM** |
| --- | --- | --- | --- |
| Optimization algorithm | Adam | Adam | Adam |
| Learning rate | 1e-3 | 1e-3 | 1e-3 |
| Maximum epochs | 500 | 500 | 500 |
| batchsize | 4 | 4 | 4 |
| Weight initialization | kaiming_uniform | kaiming_uniform | kaiming_uniform |
| Cost-sensitive learning | / | / | / |

**The hyperparameter settings of belt conveyor fault data classification networks with real-world data (in Fig. 7c):**

|  | **CNN** | **ResNet34** | **CNN-BiLSTM** |
| --- | --- | --- | --- |
| Optimization algorithm | Adam | Adam | Adam |
| Learning rate | 1e-3 | 1e-3 | 1e-3 |
| Maximum epochs | 500 | 500 | 500 |
| Batchsize | 4 | 4 | 4 |
| Weight initialization | kaiming_uniform | kaiming_uniform | kaiming_uniform |
| Cost-sensitive learning | Yes | Yes | Yes |
| Weights of classes | 0.2 (class 0);  1 (class 1);  1 (class 1) | 0.2 (class 0);  1 (class 1);  1 (class 1) | 0.2 (class 0);  1 (class 1);  1 (class 1) |

In this case, the number of normal data samples (450) was significantly larger than that of the fault data samples (two classes, 10 samples each). Therefore, when training the classifier using a small amount of real-world event data, we applied simple data augmentation strategies to mitigate this imbalance:

First, each fault sample was directly duplicated 44 times to match the number of normal samples.

Second, a basic cost-sensitive learning method was adopted to reduce the influence of the large number of normal samples during training. The core idea of cost-sensitive learning is to incorporate different classification costs for each class, encouraging the model to pay more attention to those classes with higher costs, thus improving performance. In our implementation, we assigned weights of 0.2, 1, and 1 to the normal class and the two fault classes, respectively, during the loss computation. This weighting ensures that the model focuses more on the two fault classes during training.

## 4.2 Supplementary data of classification networks

Figure S18 Accuracy of the classification networks trained with 50 RWD samples per class over 100 trials.

Unlike PIGN-generated data, RWD contains more diverse signals, and its distribution may differ from that of the test data. As a result, with a small RWD dataset, the distribution of the selected data can significantly affect the training outcome. We randomly selected 50 samples per class from the public dataset and trained the network using them. We repeated this experiment 100 times, where 50 samples per class were randomly selected for training in each trial. With 100 random selections, each choosing 50 samples from 2040 data points, the data distribution should be well covered. The classification accuracy results of the 100 independent trials are shown in Figure S18. Based on the above 100 test results, the highest accuracy achieved by CNN, Res, and CNN-BiLSTM with 50 training samples per class is 77.1%, 58.2%, and 65.7%, respectively. These results are generally consistent with those shown in the main text.

Figure S19 The training loss function and testing accuracy curves of different networks with initial or denoised data in the four classification training of the belt conveyor simulation test site.

Figure S20 Classification results of initial data and denoised data in different sizes of training sets for belt conveyor simulation testing site. The classification network is CNN.

As the training dataset increases, the classification performance of the network trained on denoised data consistently outperforms that of the network trained on initial data, as shown in Figure S20. The network trained by the denoised data has higher classification accuracy for the normal data, which means that the noise-removal net can effectively eliminate the background noise similar to the fault signal in the normal data.

Figure S21 Detailed classification performance of Figure 6b.

# 5. Statistical significance tests

## 5.1 Statistical test methods

We chose McNemar’s test (McNemar's test - Wikipedia) to evaluate whether the differences in classification accuracy between two neural networks on the same test samples are statistically significant. The reason is as follows: (1) Although McNemar’s test was originally designed for binary classification, it can still be applied to multi-class classification problems and has been used in the machine learning community to compare the performance of two classifiers (Nakata, N., & Siina, T. (2023). Ensemble learning of multiple models using deep learning for multiclass classification of ultrasound images of hepatic masses. Bioengineering, 10(1), 69. Table 2. Contingency table used for the McNemar’s test to compare two classifiers.). (2) McNemar’s test requires a minimum sample size of more than 25 (The Ultimate Guide to McNemar's Test). Although the number of test samples is limited in some of our application scenarios, it still satisfies this requirement.

When applying McNemar’s test to compare two classifiers, the test yields a chi-square statistic (chi2) and a p-value. The chi2 statistic measures the degree of difference in the classifiers’ error patterns: a larger value indicates a stronger discrepancy. The p-value represents the probability of observing such a difference under the null hypothesis that the two classifiers exhibit no performance difference on the same dataset. Importantly, statistical significance is determined by the p-value rather than the absolute magnitude of chi2. In general, p < 0.05 indicates a significant performance difference between the classifiers; p between 0.05 and 0.10 suggests a possible difference; and p > 0.10 implies no statistically detectable difference.

For the comparison of Precision and Recall, we employed a bootstrapping procedure. The reason is that Precision and Recall are ratio-based metrics and therefore not suitable for direct evaluation with McNemar’s test. Bootstrapping has been used for assessing differences in Precision and Recall between classifiers (Nava-Muñoz, S., Graff Guerrero, M., & Escalante, H. J. (2023, June). Comparison of classifiers in challenge scheme. In Mexican Conference on Pattern Recognition (pp. 89-98). Cham: Springer Nature Switzerland.), making it an appropriate choice for our analysis.

For two classifiers A and B, the Bootstrap analysis produces results of the form:

Precision Difference 95% Confidence Interval (CI): [L, R]

Recall Difference 95% CI: [L, R],

where Precision Difference = Precision_A – Precision_B and Recall Difference = Recall_A − Recall_B.

If L > 0, the metric for classifier A is significantly higher than that of B, and a larger L indicates a stronger advantage for A.

If R < 0, A performs significantly worse than B, with smaller R indicating a stronger advantage for B.

If L < 0 < R, the difference between A and B is not statistically significant; in this case, if R is greater than |L|, classifier A may perform slightly better, though not significantly.

## 5.2 Statistical significance tests results

The detailed results and corresponding discussions are provided below.

**Results in Fig. 3c**

|  | **PIGN**  Number of samples per class in training data:2040 | **RWD**  Number of samples per class in training data: 2040 | **Few-shot RWD**  Number of samples per class in training data: 50 | **PIGN**  Finetune with few-shot RWD |
| --- | --- | --- | --- | --- |
| **CNN** | ACC.: 70.8308%[68.6769, 73.0462]% (1625 samples)  Prec.: 71.7416%[69.4431, 74.0368]%  (1625 samples)  Rec.: 69.072%[67.0784, 71.0293]%  (1625 samples) | ACC.: 90.0308%[88.5538, 91.4462]%  (1625 samples)  Prec.: 89.9767%[88.4399, 91.4672]%  (1625 samples)  Rec.: 89.5143%[88.0102, 90.9778]%  (1625 samples) | ACC.: 76.6769%[74.5846, 78.7077]%  (1625 samples)  Prec.: 76.3022%[74.2191, 78.3268]%  (1625 samples)  Rec.: 76.0503%[74.0275, 78.0124]%  (1625 samples) | ACC.: 82.7692%[80.9231, 84.6154]%  (1625 samples)  Prec.: 82.9048%[80.981, 84.7591]%  (1625 samples)  Rec.: 81.9332%[80.0873, 83.7478]%  (1625 samples) |
| **ResNet34** | ACC.: 72.8%[70.6462, 74.9538]%  (1625 samples)  Prec.: 73.2609%[70.9803, 75.5129]%  (1625 samples)  Rec.: 72.0744%[69.9661, 74.195]%  (1625 samples) | ACC.: 84.2462%[82.4615, 86]%  (1625 samples)  Prec.: 84.2044%[82.3848, 85.9543]%  (1625 samples)  Rec.: 84.2523%[82.5135, 85.967]%  (1625 samples) | ACC.: 54.9538%[52.4923, 57.4154]%  (1625 samples)  Prec.: 49.1147%[45.9538, 52.2464]%  (1625 samples)  Rec.: 52.8458%[50.9536, 54.7045]%  (1625 samples) | ACC.: 82.7692%[80.9231, 84.6154]%  (1625 samples)  Prec.: 82.9048%[81.0147, 84.7313]%  (1625 samples)  Rec.: 81.9332%[80.1098, 83.7363]%  (1625 samples) |
| **CNN-BiLSTM** | ACC.: 72.4308%[70.2769, 74.5231]%  (1625 samples)  Prec.: 78.0543%[76.0044, 80.0601]%  (1625 samples)  Rec.: 70.6808%[68.7474, 72.628]%  (1625 samples) | ACC.: 96.6769%[95.8154, 97.5385]%  (1625 samples)  Prec.: 96.7268%[95.8446, 97.5753]%  (1625 samples)  Rec.: 96.5215%[95.5991, 97.4185]%  (1625 samples) | ACC.: 63.9385%[61.6, 66.2769]%  (1625 samples)  Prec.: 67.208%[64.8395, 69.5662]%  (1625 samples)  Rec.: 63.7863%[61.4585, 66.0722]%  (1625 samples) | ACC.: 86.2154%[84.4923, 87.8769]%  (1625 samples)  Prec.: 85.882%[84.1125, 87.5953]%  (1625 samples)  Rec.: 85.5864%[83.8664, 87.2719]%  (1625 samples) |

**Statistical test comparison of Fig. 3c**

**CNN-PIGN Vs CNN-RWD:**

McNemar chi2 = 262.8288, p-value = 0.00000

Precision Difference 95% CI: [-0.1552, -0.1189]

Recall Difference 95% CI: [-0.1684, -0.1384]

The p-value of 0 indicates that CNN-PIGN and CNN-RWD exhibit a statistically significant difference. The Bootstrap analysis further shows that CNN-RWD significantly outperforms CNN-PIGN. This result is expected, as CNN-RWD is trained on a large amount of real data, whereas CNN-PIGN is trained on data generated by the PIGN model.

**CNN-PIGN-Finetune Vs CNN-RWD:**

McNemar chi2 = 116.0085, p-value = 0.00000

Precision Difference 95% CI: [-0.0634, -0.0432]
Recall Difference 95% CI: [-0.0665, -0.0476]

The p-value of 0 indicates that CNN-PIGN-Finetune and CNN-RWD show a statistically significant difference. The Bootstrap analysis further demonstrates that CNN-RWD significantly outperforms CNN-PIGN-Finetune. This outcome is expected, as CNN-RWD is trained on a large amount of real data, whereas CNN-PIGN-Finetune is only fine-tuned with a small amount of real data on top of a model initially trained using PIGN-generated data.

**CNN-PIGN-Finetune Vs CNN-Few-shot-RWD:**

McNemar chi2 = 97.0101, p-value = 0.00000

Precision Difference 95% CI: [0.0400, 0.0596]
Recall Difference 95% CI: [0.0358, 0.0527]

The p-value of 0 indicates a statistically significant difference between CNN-PIGN-Finetune and CNN-Few-shot-RWD. The Bootstrap analysis further shows that CNN-PIGN-Finetune significantly outperforms CNN-Few-shot-RWD. This demonstrates that, given the same limited amount of real training data, the model trained with PIGN-generated data achieves better classification performance, highlighting the effectiveness of the PIGN-generated data.

**ResNet34-PIGN Vs ResNet34-RWD:**

McNemar chi2 = 184.0054, p-value = 0.00000

Precision Difference 95% CI: [-0.0942, -0.0703]
Recall Difference 95% CI: [-0.1033, -0.0797]

The p-value of 0 indicates a statistically significant difference between ResNet34-PIGN and ResNet34-RWD. The Bootstrap analysis further shows that ResNet34-RWD significantly outperforms ResNet34-PIGN. This result is expected, as ResNet34-RWD is trained on a large amount of real data, whereas ResNet34-PIGN is trained on data generated by the PIGN model.

**ResNet34-PIGN-Finetune Vs ResNet34-RWD:**

McNemar chi2 = 2.8138, p-value = 0.09346

Precision Difference 95% CI: [-0.0228, 0.0034]
Recall Difference 95% CI: [-0.0290, -0.0058]

The p-value of 0.09346 indicates that ResNet34-PIGN-Finetune and ResNet34-RWD show a possible difference. The Bootstrap analysis further demonstrates that the difference between ResNet34-PIGN-Finetune and ResNet34-RWD is not statistically significant. This result shows that, under the ResNet34 architecture, the model fine-tuned with a small amount of real-world data on top of PIGN-generated data can achieve performance comparable to that of a model trained with a large amount of real-world data. This demonstrates the effectiveness of using PIGN-generated data for network training.

**ResNet34-PIGN-Finetune Vs ResNet34 -Few-shot-RWD:**

McNemar chi2 = 65.5792, p-value = 0.00000

Precision Difference 95% CI: [0.1327, 0.1800]
Recall Difference 95% CI: [0.0957, 0.1268]

The p-value of 0 indicates a statistically significant difference between ResNet34-PIGN-Finetune and ResNet34-Few-shot-RWD. The Bootstrap analysis further shows that ResNet34-PIGN-Finetune significantly outperforms ResNet34-Few-shot-RWD. This demonstrates that, given the same limited amount of real training data, the model trained with PIGN-generated data achieves better classification performance, highlighting the effectiveness of the PIGN-generated data.

**CNN-BiLSTM-PIGN Vs CNN-BiLSTM -RWD:**

McNemar chi2 = 376.7049, p-value = 0.00000

Precision Difference 95% CI: [-0.1560, -0.1246]
Recall Difference 95% CI: [-0.2088, -0.1791]

The p-value of 0 indicates a statistically significant difference between CNN-BiLSTM-PIGN and CNN-BiLSTM-RWD. The Bootstrap analysis further shows that CNN-BiLSTM-RWD significantly outperforms CNN-BiLSTM-PIGN. This result is expected, as CNN-BiLSTM-RWD is trained on a large amount of real data, whereas ResNet34-PIGN is trained on data generated by the PIGN model.

**CNN-BiLSTM-PIGN-Finetune Vs CNN-BiLSTM-RWD:**

McNemar chi2 = 168.0059, p-value = 0.00000

Precision Difference 95% CI: [-0.0929, -0.0697]
Recall Difference 95% CI: [-0.0932, -0.0706]

The p-value of 0 indicates that CNN-BiLSTM-PIGN-Finetune and CNN-BiLSTM-RWD show a statistically significant difference. The Bootstrap analysis further demonstrates that CNN-BiLSTM-RWD significantly outperforms CNN-BiLSTM-PIGN-Finetune. This outcome is expected, as CNN-BiLSTM-RWD is trained on a large amount of real data, whereas CNN-BiLSTM-PIGN-Finetune is only fine-tuned with a small amount of real data on top of a model initially trained using PIGN-generated data.

**CNN-BiLSTM-PIGN-Finetune Vs CNN-BiLSTM-Few-shot-RWD:**

McNemar chi2 = 344.7646, p-value = 0.00000

Precision Difference 95% CI: [0.1272, 0.1532]
Recall Difference 95% CI: [0.1490, 0.1781]

The p-value of 0 indicates a statistically significant difference between CNN-BiLSTM-PIGN-Finetune and CNN-BiLSTM-Few-shot-RWD. The Bootstrap analysis further shows that CNN-BiLSTM-PIGN-Finetune significantly outperforms CNN-BiLSTM-Few-shot-RWD. This demonstrates that, given the same limited amount of real training data, the model trained with PIGN-generated data achieves better classification performance, highlighting the effectiveness of the PIGN-generated data.

**Results in Fig. 6b**

|  | **Initial** | **Denoised** | **Statistical test comparison:**  **Initial vs Denoised** |
| --- | --- | --- | --- |
| **CNN** | Acc.: 68.3333%[60, 76.6667]%  (120 samples)  Prec.: 74.359%[67.2057, 80.7858]%  (120 samples)  Rec.: 68.3333%[60.7721, 75.7787]%  (120 samples) | Acc.: 76.6667%[68.3333, 84.1667]%  (120 samples)  Prec.: 78.7212%[71.5879, 85.4929]%  (120 samples)  Rec.: 76.6667%[69.2076, 83.7183]%  (120 samples) | McNemar chi2 = 4.5000, p-value = 0.03389  Precision Difference 95% CI: [-0.1030, 0.0203] Recall Difference 95% CI: [-0.1484, -0.0203] |
| **ResNet34** | Acc.: 63.3333%[55, 71.6667]%  (120 samples)  Prec.: 63.8713%[55.2342, 72.2614]%  (120 samples)  Rec.: 63.3333%[55.4719, 71.1169]%  (120 samples) | Acc.: 67.5%[59.1667, 75.8333]%  (120 samples)  Prec.: 65.9601%[57.1049, 74.5659]%  (120 samples)  Rec.: 67.5%[59.9761, 75]%  (120 samples) | McNemar chi2 = 0.6400, p-value = 0.42371  Precision Difference 95% CI: [-0.1033, 0.0593] Recall Difference 95% CI: [-0.1151, 0.0286] |
| **CNN-BiLSTM** | Acc.: 74.1667%[65.8333, 81.6667]%  (120 samples)  Prec.: 74.6057%[66.5979, 82.0553]%  (120 samples)  Rec.: 74.1667%[66.5726, 81.2816]%  (120 samples) | Acc.: 75.8333%[68.3333, 83.3333]%  (120 samples)  Prec.: 76.6627%[69.395, 83.7191]%  (120 samples)  Rec.: 75.8333%[68.487, 83.0721]%  (120 samples) | McNemar chi2 = 0.0833, p-value = 0.77283  Precision Difference 95% CI: [-0.0764, 0.0325] Recall Difference 95% CI: [-0.0698, 0.0373] |

From the statistical comparison between CNN-initial and CNN-denoised, the p-value is 0.034, which is below 0.05, indicating a statistically significant difference in classification accuracy between the two networks. The Bootstrap analysis further shows that the two networks do not differ significantly in precision, whereas the recall of the CNN trained on denoised data is significantly higher than that of the CNN trained on raw data. This demonstrates that applying the noise-removal network effectively improves the final classification performance, confirming that the noise-removal network can successfully suppress noise while preserving event-related features.

For the ResNet34 and CNN-BiLSTM architectures, the classification performance between models trained on raw data and those trained on denoised data does not show statistically significant differences. In these more complex network architectures, the performance improvement brought by denoising is less pronounced. However, these results at least indicate that the noise-removal network does not remove essential event features of the initial data or degrade the classification performance of the subsequent classifiers.

**Results in Fig. 7c**

|  | **CNN-BiLSTM-PIGN** | **CNN-BiLSTM-RWD** | **CNN-BiLSTM-PIGN-Finetune** | **Artificially designed algorithm** |
| --- | --- | --- | --- | --- |
| **Three-category** | Acc.: 85.6492%[82.2323, 88.8383]% (439 samples)  Prec.: 86.0357%[82.7386, 89.2389]%  (439 samples)  Rec.: 85.1185%[81.7597, 88.3576]% (439 samples) | Acc.: 71.2984%[66.9704, 75.3986]%  (439 samples)  Prec.: 73.0261%[68.6321, 77.2985]%  (439 samples)  Rec.: 70.4688%[66.4974, 74.3479]%  (439 samples) | Acc.: 88.3827%[85.1936, 91.344]%  (439 samples)  Prec.: 88.3562%[85.2116, 91.3433]%  (439 samples)  Rec.: 88.1525%[85.0212, 91.1407]%  (439 samples) |  |
| **two-category** | Acc.: 91.7995%[89.0661, 94.3052]%  (439 samples)  Prec.: 90.6314%[87.682, 93.3722]%  (439 samples)  Rec.: 92.7561%[90.3128, 94.9943]%  (439 samples) | Acc.: 82.2323%[78.5877, 85.877]%  (439 samples)  Prec.: 81.9632%[78.4538, 85.3379]%  (439 samples)  Rec.: 84.5766%[81.2702, 87.6681]%  (439 samples) | Acc.: 93.3941%[90.8884, 95.672]%  (439 samples)  Prec.: 92.5002%[89.7205, 95.0605]%  (439 samples)  Rec.: 93.4625%[90.9309, 95.734]%  (439 samples) | Acc.: 86.5604%[83.3713, 89.7494]%  (439 samples)  Prec.: 86.3499%[83.2724, 89.3519]%  (439 samples)  Rec.: 89.3284%[86.833, 91.7299]%  (439 samples) |

**Statistical test comparison of Fig.7c**

**CNN-BiLSTM-PIGN-Three-category Vs CNN-BiLSTM-RWD-Three-category:**

McNemar chi2 = 47.4568, p-value = 0.00000

Precision Difference 95% CI: [0.0685, 0.1274]
Recall Difference 95% CI: [0.0826, 0.1373]

Based on the p-value of 0, CNN-BiLSTM-PIGN-Three-category and CNN-BiLSTM-RWD-Three-category exhibit a statistically significant difference. The Bootstrap analysis further shows that CNN-BiLSTM-PIGN-Three-category performs significantly better than CNN-BiLSTM-RWD-Three-category. This indicates that the network trained on PIGN-generated data outperforms the one trained on limited real-world data, demonstrating the effectiveness of the PIGN-generated data.

**CNN-BiLSTM-PIGN-Finetune-Three-category Vs CNN-BiLSTM-RWD-Three-category:**

McNemar chi2 = 65.9759, p-value = 0.00000

Precision Difference 95% CI: [0.0881, 0.1426]
Recall Difference 95% CI: [0.1057, 0.1604]

Based on the p-value of 0, CNN-BiLSTM-PIGN-Finetune-Three-category and CNN-BiLSTM-RWD-Three-category exhibit a statistically significant difference. The Bootstrap analysis further shows that CNN-BiLSTM-PIGN-Finetune-Three-category performs significantly better than CNN-BiLSTM-RWD-Three-category.

**CNN-BiLSTM-PIGN-Two-category Vs Artificially designed algorithm:**

McNemar chi2 = 14.6667, p-value = 0.00013

Precision Difference 95% CI: [0.0101, 0.0327]
Recall Difference 95% CI: [0.0057, 0.0283]

Based on the p-value of 0.00013, CNN-BiLSTM-PIGN-Two-category and Artificially designed algorithm exhibit a statistically significant difference. The Bootstrap analysis further shows that CNN-BiLSTM-PIGN-Two-category performs significantly better than Artificially designed algorithm.

**CNN-BiLSTM-PIGN-Two-category Vs CNN-BiLSTM-RWD-Two-category:**

McNemar chi2 = 40.0238, p-value = 0.00000

Precision Difference 95% CI: [0.0312, 0.0560]
Recall Difference 95% CI: [0.0291, 0.0534]

Based on the p-value of 0, CNN-BiLSTM-PIGN-Two-category and CNN-BiLSTM-RWD- Two-category exhibit a statistically significant difference. The Bootstrap analysis further shows that CNN-BiLSTM-PIGN-Two-category performs significantly better than CNN-BiLSTM-RWD-Two-category. This indicates that the network trained on PIGN-generated data outperforms the one trained on limited real-world data, demonstrating the effectiveness of the PIGN-generated data.

**CNN-BiLSTM-PIGN-Finetune-Two-category Vs CNN-BiLSTM-RWD-Two-category:**

McNemar chi2 = 47.0204, p-value = 0.00000

Precision Difference 95% CI: [0.0397, 0.0660]
Recall Difference 95% CI: [0.0332, 0.0561]

Based on the p-value of 0, CNN-BiLSTM-PIGN-Finetune-Two-category and CNN-BiLSTM-RWD-Two-category exhibit a statistically significant difference. The Bootstrap analysis further shows that CNN-BiLSTM-PIGN-Finetune-Two-category performs significantly better than CNN-BiLSTM-RWD-Two-category.

# 6. Experiment

## 6.1 Experimental details of public datasets

**Experimental settings for all data in the public dataset are as follows:**

1. To ensure diversity, each type of event in the dataset involved at least two different participants.

2. All data were collected indoors, within approximately 50 meters from the fiber end. Two different fiber lengths were used: 5 km and 10 km.

3. To enhance the diversity within each event class, data were collected across multiple days and during various times of day, including morning, afternoon, and night.

4. The sampling rate was 10 ${Msamples s}^{-1}$, with a spatial resolution of approximately 10 meters.

5. Training and testing datasets were randomly split from the collected data.

The total duration of 1,000 time sample points is approximately 10 seconds. The following content is the reason. As shown in Fig. 3a (fast walk and slow walk, also reproduced below for ease of reference), we observed that the fast walk segment contains 36 periodic cycles within the recording window, corresponding to 36 steps.

Considering that the human maximum step frequency is around 5 Hz (e.g., elite sprinters exhibit an average step frequency of approximately 4.17 Hz during the 100-m dash, as shown in the Reference: Morin, J. B., Bourdin, M., Edouard, P., Peyrot, N., Samozino, P., & Lacour, J. R. (2012). Mechanical determinants of 100-m sprint running performance. *European journal of applied physiology*, *112*(11), 3921-3930. Table 1.), a relatively reasonable duration for such a walk data segment is about 10 seconds, which corresponds to a step frequency of approximately 3.6 Hz (already close to the lower bound of sprinting cadence of elite sprinters, ~3.8 Hz from the Reference *European journal of applied physiology*, *112*(11), 3921-3930.). Based on this reasonable total duration of 10 s and the total number of points (1000), we estimated the sampling frequency to be around 100 Hz. This estimated sampling frequency was then used to generate the time-domain features for both shake and walk events.

**The experimental details specific to the walk event are as follows:**

1. The participants walked close to the fiber, at a distance of approximately 0.2 meters.

2. The fiber was laid directly on the floor.

3. At least three individuals participated in the walk data collection.

4. The walk event was recorded over four days, with the number of samples (train + test) collected each day being: 4, 1061, 785, and 600, respectively.

5. Participants performed slow and fast walking.

**The experimental details specific to the shake event are as follows:**

1. The fiber was fixed in a U-shaped, bent configuration onto an iron cage, with a total length of approximately 15–20 meters.

2. Tests were conducted at various locations across the entire deployed fiber range, covering a total of 10 different positions.

3. The shake data were collected over three days, with the number of samples (train + test) collected on each day being 1419, 531, and 778, respectively.

## 6.2 DAS system in the belt conveyor fault monitoring

In the application of belt conveyor fault monitoring, the DAS system is based on TGD-OFDR. DAS interrogator (ixDAS-4000) is provided by Ningbo AllianStream Photonics Technology Co., Ltd. When conducting experiments, the sampling rate is set to 30 kHz.

The specifications of DAS are as follows:

| **Model** | **ixDAS-4000** | |
| --- | --- | --- |
| **Measurand** | Strain / Strain rate | |
| **Sensing Range** | 60 km (maximum) | |
| **Spatial Resolution**  **(Gauge Length)** | 3.6 m (minimum) | |
| **Spatial Sampling Interval** | 1 gauge length | |
| **Sampling Rate** | 100 kSps (maximum) | |
| **Response Frequency** | 1 Hz ~ 20 kHz | |
| **Self-Noise Level ^1^** | @ 1km | @ 50km |
|  | 10 pε $\sqrt{Hz}^{-1}$ @20 Hz  5 pε $\sqrt{Hz}^{-1}$ @100 Hz | 40 pε $\sqrt{Hz}^{-1}$ @20 Hz  15 pε $\sqrt{Hz}^{-1}$ @100 Hz |
| **Dynamic Range** | ≥ 95 dB @ 10 Hz | |
| **Fiber and Connector** | SMF / MMF / Specialty fiber cable, FC/APC | |
| **Average Emission Power** | ≤ 0.5 mW (laser safety 1 class) | |
| **Fiber Quality Monitoring** | A real-time backscattering relative intensity profile output provided | |
| **Channels** | 1 | |
| **Data Interface** | RJ45 (1000M Ethernet) | |
| **Operation Temperature** | - 20 ℃ to 50 ℃ | |
| **Size** | 429 mm (W) × 483 mm (D) × 86 mm (H) | |
| **Power Supply** | 100~240 VAC, 60 W | |

## 6.3 Sites of belt conveyor


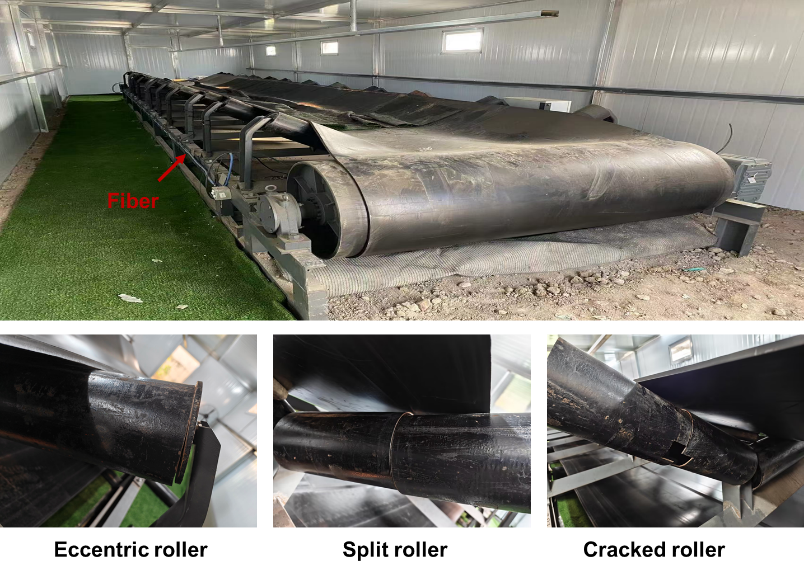


Figure S22 Belt conveyor simulation test site and three types of fault rollers.


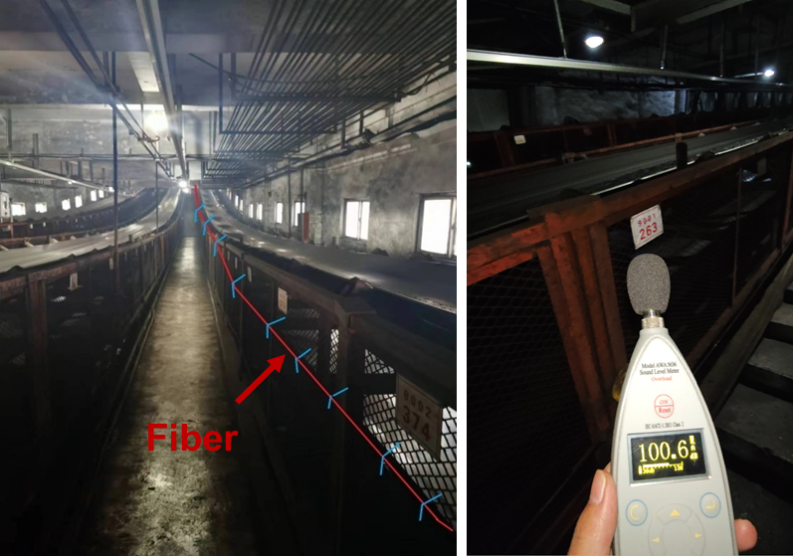


Figure S23 Belt conveyor at coal mine site and on-site environmental sound noise measurement result.

## 6.4 Experimental details of belt conveyor fault monitoring

**Belt conveyor simulation test site**

The total fiber length was approximately 250 meters, with the final 50 meters of the fiber deployed along a 13-meter-long conveyor system. The fiber was laid in a straight line along the frame of the conveyor on both sides. Some fiber loops were introduced when transitioning from one side of the conveyor to the other. Faulty rollers were installed at three main locations, approximately 4 m, 8 m, and 16 m from the fiber end.

The training and testing data were also randomly split. The three types of roller faults were collected over three separate days, with each day dedicated to one fault type. For each fault type, we collected data under three different fault positions on the conveyor and three belt speeds, introducing diversity within the same fault class. Although data for each fault type were collected on the same day, the variations in fault location and operational conditions (e.g., belt speed) provided diversity. Due to the time-consuming and labor-intensive process of roller disassembly and installation, data collection for a single fault type typically spanned from 9 a.m. to 9 p.m. In contrast, normal (non-fault) data were collected over three days, with 15 samples recorded per day.

**Coal mine site**

The conveyor was approximately 400 meters long, and the total fiber length was about 1 km. Fiber was deployed on both sides of the conveyor in a straight-line manner as much as possible, with occasional fiber loops introduced due to practical constraints during deployment. The timing and positions of faulty rollers in this field site are listed as table S1.

Table S1 Roller failure record of the coal mine site

| **Time** | **Fault roller group number** |
| --- | --- |
| 2024.04.04. | 13 (ND); 243 (ND) |
| 2024.04.15. | 305 (SD) |
| 2024.04.16. | 196 (SD); 105 (ND) |
| 2024.04.22. | 7 (NU) |
| 2024.04.25 | 57 (SD) |
| 2024.05.04. | 147 (SD) |
| 2024.05.06. | 169 (SD) |
| 2024.05.19. | 64 (SU) |
| 2024.05.21. | 178 (ND) |
| 2024.05.26. | 58 (U) |
| 2024.05.29. | 218 (U) |
| 2024.05.30. | 252 (SD) |
| 2024.06.04. | 147 (ND) |
| 2024.06.06. | 115 (U) |
| 2024.06.12. | 46 (D) |
| 2024.06.19. | 323 (SD) |
| 2024.06.20. | 218 (ND) |
| 2024.07.03 | 25 (ND) |
| 2024.07.18. | 82 (ND); 218 (ND); 58 (D) |
| 2024.07.19. | 169 (ND) |
| 2024.07.26. | 95 (ND);216 (SD) |
| 2024.07.29. | 64 (U) |
| 2024.07.31 | 122 (ND) |

In this coal mine field, each roller group was indexed sequentially. Each group typically included at least two upper and two lower rollers, located on both sides of the conveyor (referred to as the South and North sides in the coal mine). While the general distance between two adjacent roller groups is about 1.2 meters, this varied due to installation differences. As a result, the fault roller group number can be used to approximate the spatial location of the disturbance.

In the above table provided, the symbols “S” and “N” denote the south-facing and north-facing sides of the conveyor, respectively, while “D” and “U” refer to lower and upper rollers, respectively. It is worth noting that due to variations in the quality and diligence of manual inspections conducted by workers from coal mine, some of the fault feedback and labeling may be incomplete.

For the coal mine site, the fault data were not used during training. In our framework, training requires background (normal) data. To this end, we randomly selected 450 samples of DAS data from normal rollers at various times and locations on April 2, 2024, to serve as training data for normal class. The training data for fault events was generated by PIGN. As for the testing data, fault samples and their associated timestamps and roller positions are provided in the table S1. The first fault data used for test data occurred on April 4, 2024. A total of 23 faults occurred until July 31, 2024. The normal samples in the test set were randomly selected from DAS data collected between April 4, 2024, and July 31, 2024, specifically from locations not reported to have faults.

**Training and testing dataset**

For the RWD experiment, there were in total 450 samples for each of the three classes used to train the classification network. The RWD experiment and the PIGN experiment shared the same testing dataset, which consisted entirely of real-world data: 159 normal samples, 137 fault-class-1 samples, and 143 fault-class-2 samples (439 testing samples in total).

Accordingly, for the RWD experiment, the dataset composition was as follows:

Training data: 450 normal RWD samples; 450 fault-class-1 samples (10 original fault-class-1 RWD samples + 440 augmented samples); 450 fault-class-2 samples (10 original fault-class-2 RWD samples + 440 augmented samples).

Testing data: 159 normal RWD samples; 137 fault-class-1 RWD samples; 143 fault-class-2 RWD samples.

All real-world training and testing samples were collected at the coal-mine site. Each sample was acquired at a different time, with a minimum interval of at least 2 minutes, and typically around 20 minutes between acquisitions for fault data. The same acquisitions temporal spacing applies to the samples used as RWD training data and RWD testing data.

For the PIGN experiment, the division between training and testing data was as follows:

Training data: 450 normal RWD samples; 450 fault-class-1 PIGN generated samples; 450 fault-class-2 PIGN generated samples.

Testing data: 159 normal RWD samples; 137 fault-class-1 RWD samples; 143 fault-class-2 RWD samples.

# 7. Supplementary data and enlarged figures related to the main text

**Enlarged version of Figure 4b:**

Figure S24 Enlarged version of Figure 4b

As shown in Figure S24, each roller corresponds to a distinct spatial location, and the operational states of the rollers are nearly independent of one another. As a result, the signals at different spatial positions do not interfere with each other, which can be observed in Figure S24. Due to this property, it becomes necessary to extract the temporal signal at each spatial location and perform time-frequency analysis to assess the state of individual rollers.

**Enlarged versions of Figure 4d and Figure 4f:**

Figure S25 Enlarged version of Figure 4d and 4f.

As shown in Figure S25, it can be seen that after noise removal, the fault feature originally buried in the background noise at the 9-second mark is effectively extracted, with an amplitude more than twice that of the noise.

# Reference

1. Mooney, Melvin. "A theory of large elastic deformation." *Journal of applied physics* 11.9 (1940): 582-592.
2. Dian Chen.” Research of High-performance Fiber-optic Distributed Acoustic Sensor Based on Time-gated Digital Optical Frequency Domain Reflectometry”. Diss. Shanghai Jiao Tong University, 2020.
3. Jacobsen, Lydik S. "An approximate solution of the steady forced vibration of a system of one degree of freedom under the influence of various types of dam**." *Bulletin of the Seismological Society of America* 20.3 (1930): 196-223.
4. Bao, Zhijun, et al. "Experimental Study on the Law of Human Walking Speed." Research and Exploration in Laboratory, vol. 39, no. 4, 2000, pp. 39-42.
5. Chen, Jiayu, et al. "Fault diagnosis of rotating machinery: A review and bibliometric analysis." *Ieee Access* 8 (2020): 224985-225003.
6. Xu, Zhi-Qin John, Yaoyu Zhang, and Yanyang Xiao. "Training Behavior of Deep Neural Network in Frequency Domain." Neural Information Processing, edited by T. Gedeon, K. Wong, and M. Lee, Springer, Cham, 2019. Lecture Notes in Computer Science, vol. 11953, pp. 264-274.
7. Ciabattoni, L., Ferracuti, F., Freddi, A., & Monteriu, A. (2017). Statistical spectral analysis for fault diagnosis of rotating machines. IEEE Transactions on Industrial Electronics, 65(5), 4301-4310.
8. Lee, S. K., & White, P. R. (1997). Higher-order time–frequency analysis and its application to fault detection in rotating machinery. Mechanical Systems and Signal Processing, 11(4), 637-650.
9. Al-Badour, F., Sunar, M., & Cheded, L. (2011). Vibration analysis of rotating machinery using time–frequency analysis and wavelet techniques. Mechanical Systems and Signal Processing, 25(6), 2083-2101.
